# Local states of chromatin compaction at transcription start sites control transcription levels

**DOI:** 10.1093/nar/gkab587

**Published:** 2021-07-07

**Authors:** Satoru Ishihara, Yohei Sasagawa, Takeru Kameda, Hayato Yamashita, Mana Umeda, Naoe Kotomura, Masayuki Abe, Yohei Shimono, Itoshi Nikaido

**Affiliations:** Fujita Health University School of Medicine, Toyoake, Aichi 470-1192, Japan; Laboratory for Bioinformatics Research, RIKEN Center for Biosystems Dynamics Research, Wako, Saitama 351-0198, Japan; Functional Genome Informatics, Medical Research Institute, Tokyo Medical and Dental University, Bunkyo-ku, Tokyo 113-8510, Japan; Laboratory for Bioinformatics Research, RIKEN Center for Biosystems Dynamics Research, Wako, Saitama 351-0198, Japan; Graduate School of Science, Hiroshima University, Higashi-Hiroshima, Hiroshima 739-8526, Japan; Graduate School of Engineering Science, Osaka University, Toyonaka, Osaka 560-8531, Japan; Laboratory for Bioinformatics Research, RIKEN Center for Biosystems Dynamics Research, Wako, Saitama 351-0198, Japan; Fujita Health University School of Medicine, Toyoake, Aichi 470-1192, Japan; Graduate School of Engineering Science, Osaka University, Toyonaka, Osaka 560-8531, Japan; Fujita Health University School of Medicine, Toyoake, Aichi 470-1192, Japan; Laboratory for Bioinformatics Research, RIKEN Center for Biosystems Dynamics Research, Wako, Saitama 351-0198, Japan; Functional Genome Informatics, Medical Research Institute, Tokyo Medical and Dental University, Bunkyo-ku, Tokyo 113-8510, Japan; Master's/Doctoral Program in Life Science Innovation (Bioinformatics), Degree Programs in Systems and Information Engineering, Graduate School of Science and Technology, University of Tsukuba, Wako, Saitama 351-0198, Japan

## Abstract

The ‘open’ and ‘compact’ regions of chromatin are considered to be regions of active and silent transcription, respectively. However, individual genes produce transcripts at different levels, suggesting that transcription output does not depend on the simple open-compact conversion of chromatin, but on structural variations in chromatin itself, which so far have remained elusive. In this study, weakly crosslinked chromatin was subjected to sedimentation velocity centrifugation, which fractionated the chromatin according to its degree of compaction. Open chromatin remained in upper fractions, while compact chromatin sedimented to lower fractions depending on the level of nucleosome assembly. Although nucleosomes were evenly detected in all fractions, histone H1 was more highly enriched in the lower fractions. H1 was found to self-associate and crosslinked to histone H3, suggesting that H1 bound to H3 interacts with another H1 in an adjacent nucleosome to form compact chromatin. Genome-wide analyses revealed that nearly the entire genome consists of compact chromatin without differences in compaction between repeat and non-repeat sequences; however, active transcription start sites (TSSs) were rarely found in compact chromatin. Considering the inverse correlation between chromatin compaction and RNA polymerase binding at TSSs, it appears that local states of chromatin compaction determine transcription levels.

## INTRODUCTION

All physiological reactions on the genomic DNA require the binding of protein factors with various enzymatic activities to the appropriate regions of the genome. However, these reactions occasionally fail when the protein factors cannot access their target regions. In eukaryotic cells in early S-phase, replication is initiated from replication origins within genomic regions where DNA-processing enzymes, such as endonucleases, are able to access the DNA under experimental conditions (1,2). Repair and recombination in such enzyme-accessible regions also dominantly occur compared with the levels of these activities in enzyme-inaccessible regions (3–5). Similarly, more abundant transcripts are produced from genes within enzyme-accessible regions (6–8). These observations indicate that the accessibility of the genome directly controls the strength of reactions on the genome. Importantly, while replication, repair, and recombination are completed in a single round because of their all-or-nothing output, transcription is a repeated reaction because it is required to synthesize multiple RNA copies. The number of copies is determined by the frequency of the reaction, that is, the frequency of RNA polymerase (RNAP) binding to the transcription start site (TSS). Therefore, a structure that can vary in its degree of accessibility is required to allow variations in transcription levels.

The chromatin structure largely influences the accessibility of the genome. As the primary structure of chromatin, the genomic DNA is wrapped around a histone octamer to form a nucleosome (9). The nucleosomes interact with neighboring nucleosomes and/or non-histone proteins to form a higher-order structure (10). Thus, the structures organized in this step-by-step process can hide the genomic DNA from protein factors and reduce the accessibility of the genome. Via treatment with micrococcal nuclease (MNase), the nucleosome positioning along the genome has been characterized. This investigation revealed that nucleosomes are absent in the region just upstream of the TSSs of actively transcribed genes (11). It is widely accepted that such regions, known as nucleosome-free regions (NFRs), support RNAP binding (11), resulting in a direct correlation between chromatin structure and transcriptional state. However, because this level in the hierarchy of chromatin structure exists in two states, i.e., with or without nucleosomes, the ability to achieve intermediate levels of transcription might not be shown. Although NFRs are more clearly observed at the TSSs of highly transcribed genes via well-positioning of nucleosomes on both sides of the NFR (12–15), it has also been reported that the nucleosome positioning around the TSSs of genes that are uniformly transcribed in a given cell population is heterogenous (16,17). In addition to nucleosome positioning, other variables are required in the hierarchy of chromatin structure to allow tuning of the transcription levels.

X-ray structure analyses have revealed that a nucleosome interacts with another nucleosome via the basic tail of histone H4, which has affinity for an acidic patch on the surface of an adjacent nucleosome (18,19). This interaction has also been confirmed by biochemical experiments (20,21). Therefore, an array of multiple nucleosomes, known as ‘beads on a string’, is often formed via inter-nucleosomal interactions to generate a more compact structure. A 30 nm thick fiber has been historically proposed to represent this more compact structure (22). Electron microscopy analyses have revealed that *in vitro*-reconstituted nucleosome arrays fold into a 30 nm thick rod-shaped structure that has been conceptualized by two alternative models: a one-start solenoid (23) or a two-start zigzag (24). Over the last decade, the results of imaging studies have argued against the existence of the 30-nm fiber and have instead revealed granular structures that are distinct from the rod of the 30-nm fiber (25–27). Microscope imaging techniques have shown that chromatin domains consist of irregular 100–300 nm wide aggregates of nucleosomes (28–31). Such domains are thought to correspond to topologically associating domains (TADs), which were identified by Hi-C, a derivative of chromosome conformation capture (32–35). Hi-C methods also revealed that aggregates of several nucleosomes are present in mammalian cells and budding yeast (32–36). Together with live imaging analyses that showed fluctuating movement in individual nucleosomes (29,37,38), these observations suggest that a static 30-nm fiber structure is unlikely to be formed *in vivo*. How nucleosome arrays are in fact arranged remains under debate; nevertheless, it is not doubted that neighboring nucleosomes are locally compacted. Thus, such a structure must be responsible for regulating the accessibility of the genome, which ultimately controls transcription levels.

In this study, the 3D compaction state within several neighboring nucleosomes was focused on as a possible chromatin structure responsible for controlling transcription levels. Using sedimentation velocity centrifugation, chromatin from cultured cells was successfully fractionated based on its local compaction states: compact chromatin sedimented into the lower fractions, while open chromatin remained in the upper fractions. The number of nucleosomes per unit length of DNA was consistent across all fractions; however histone H1 enriched in the compact chromatin fractions. Upon crosslinking with formaldehyde (FA), histone H1 was found to interact physically with histone H3 and further histone H1 molecules in the compact chromatin fraction, suggesting that chromatin compaction results from inter-nucleosomal interactions via H1. Next-generation sequencing (NGS) of the DNA recovered from each fraction showed that nearly the entire genome was packaged into compact chromatin, with the exception of the chromatin at active TSSs, which was poorly compacted. Weakly compacted TSS chromatin more clearly correlated with transcription levels than NFR formation. Additionally, the local state of chromatin compaction appears to influence the frequency of RNAP binding, which ultimately regulates the transcription levels of individual genes.

## MATERIALS AND METHODS

### Chromatin fractionation by using sedimentation velocity centrifugation

**Figure 1.**
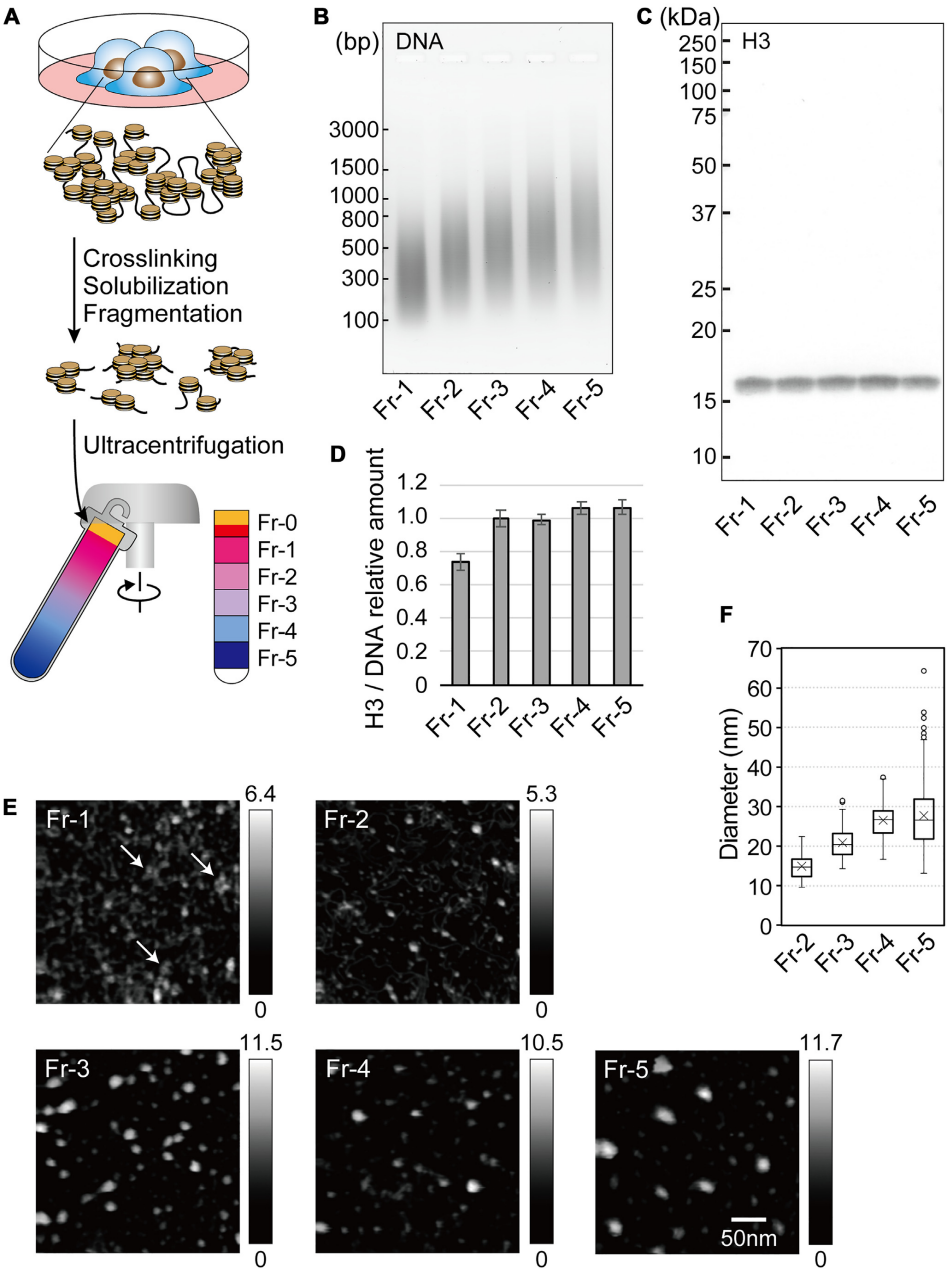
Chromatin fractionation by using sedimentation velocity centrifugation. (**A**) A schema of the fractionation method established in this study. (**B**) The size distribution of DNA fragments in fractionated chromatin. The DNA was separated by size on a 2% agarose gel and stained with SYBR Green I. Molecular weight markers are indicated by short bars. (**C**) Western blot for histone H3. Fractionated proteins were separated by size on a 10% SDS-PAGE gel, transferred to a nitrocellulose membrane, and probed with a pan anti-histone H3 antibody. Molecular weight markers are indicated by short bars. (**D**) Samples were normalized by DNA content prior to loading, and the amount of histone H3 in each fraction was calculated relative to the mean. Data were obtained from at least three independent experiments, and are represented as the mean ± SD. (**E**) HS-AFM images of the fractionated chromatin. The arrows in Fr-1 highlight several closely gathered dots. The heights of the objects are shown as a grayscale gradation ranging from 0 to a maximum (nm) in each panel. All panels are shown at the same magnification. (**F**) The diameters of the chromatin particles in each fraction.

This chromatin fractionation technique is a modification of a method we established previously (39,40), and its schema is illustrated in Figure 1A. HepG2 cells (a human hepatoma cell line obtained from the RIKEN BRC in Japan) were used in this study. HepG2 cells were cultured in a minimum essential medium with α-modification supplemented with 10% fetal bovine serum. After washing with phosphate-buffered saline (PBS), 15–90 mg (wet weight) of HepG2 cells were collected into a microtube, and the concentration was adjusted to 15 mg/ml in PBS. For the crosslinking reaction, an FA solution (#F8775, Merck) was added to the cells at a final concentration of 0.5%, and the cells were agitated at room temperature for 10 min. Following addition of glycine at a final concentration of 62.5 mM to quench the FA, the cells were washed twice with ice-cold PBS, solubilized with 250–500 μl of Tris-based sodium dodecyl sulfate (SDS) lysis buffer [TSB; 1% SDS, 50 mM Tris–HCl (pH 8.0), 10 mM EDTA and a Complete Protease Inhibitor Cocktail (#4693159001, Roche)], and then fragmented with a Sonifier liquid processor (#150D, Branson) (at level ‘2’ for 5 s 6 times on ice). After removal of the debris using a Vivaclear Mini column (#VK01P042, Sartorius), the cell extract, including the chromatin fragments, was layered onto an 11 ml sucrose gradient (20−60%), which was prepared as follows: 20% and 60% sucrose solutions in chromatin dilution buffer (CDB; 1.1% Triton X-100, 0.01% SDS, 16.7 mM Tris–HCl (pH 8.0), 1.2 mM EDTA, 167 mM NaCl and a Complete Protease Inhibitor Cocktail) were prepared, and loaded into the reservoir and mixing chambers, respectively, of a gradient maker (#GM-20, CBS Scientific). The outlet of the gradient maker was connected via a peristaltic pump to the uppermost position of the inner wall of a polyallomer centrifugation tube (#331372, Beckman Coulter). The gradient solution was delivered from the mixing chamber into the tube using the pump while mixing the sucrose solutions using a magnetic stirrer. A sample was layered on the gradient and subjected to ultracentrifugation at 38 700 rpm (RCF max: 256 000 × g) at 4°C for 16 h in a Beckman SW41Ti swing rotor. Following removal of the uppermost 1.8 ml (designated ‘Fr-0’), five 1.8 ml fractions (numbered ‘Fr-1’ to ‘Fr-5’) were collected from the top to the bottom of the tube as follows: A tip of a 1-ml micropipette, in which the dial was set to 0.9 ml, was placed at the top of the gradient. While rotating around the tube, 0.9 ml solution was gently sucked up twice and transferred to a collection tube.

### High-speed atomic force microscopy (HS-AFM) observation of fractionated chromatin

Chromatin was recovered from each fraction by immunoprecipitation with an anti-pan-histone H3 antibody (#ab1791, Abcam). Prior to the immunoprecipitation, 100 μg of the antibody was covalently conjugated to 17 mg of magnetic beads using a Dynabeads Antibody Coupling Kit (#14311D, Thermo). The H3-conjugated beads (1.2 mg of beads) were mixed with each fraction, and the mixtures were agitated at 4°C overnight. After washing three times with CDB and then once with HE (50 mM HEPES (pH 7.6), 10 mM EDTA) at 4°C for 5 min each, the chromatin was eluted in 30 μl of HEPES-based SDS lysis buffer (HSB; 1% SDS, 50 mM HEPES (pH 7.6), 10 mM EDTA, and a Complete Protease Inhibitor Cocktail). To examine nucleosome arrays in Fr-5 chromatin particles, DNA fragments in the chromatin were extended using terminal deoxynucleotidyl transferase (TdT), and observed by HS-AFM. Briefly, Fr-5 chromatin was immunoprecipitated as described above, and treated with T4 DNA polymerase (#311–02481, Nippon Gene) and exonuclease III (#2170A, Takara Bio) to repair DNA termini as described previously (41). After the chromatin was collected by re-immunoprecipitation, biotin-16-dUTP (#11093070910, Roche) was added to the DNA termini using TdT (#M828A, Promega). Following re-immunoprecipitation, biotin-incorporated DNA fragments were observed by HS-AFM (Supplementary Figure S3). Hydrophobic aggregation of chromatin particles was avoided by observing the samples under HSB containing 1% SDS, following elution from the immunoprecipitates. HS-AFM analyses were performed using a laboratory-built HS-AFM apparatus similar to previously described AFM (42). The HS-AFM was equipped with small cantilevers [*k* = 0.1–0.2 N/m, *f* = 800–1200 kHz in solution (Olympus)] and was operated in tapping mode. The AFM styli were placed on each cantilever by electron beam deposition. A sample stage made of quartz glass was placed on the z-scanner, and a 1.5 mm diameter mica disk was glued onto the sample stage. A freshly cleaved mica surface was treated with 0.1% aminosilane for 90 s. After rinsing the surface with HE, 1.5 μl sample droplets of the chromatin preparations were placed on the mica surface and incubated for 3 min. Unmodified bare mica was used to examine the number of nucleosome arrays in Fr-5 chromatin particles (Supplementary Figure S3). HS-AFM observations were performed at room temperature. To estimate the sizes of the chromatin in each fraction, the diameters of the objects in the AFM images were analyzed using SPIP image analysis software (Image Metrology) and Origin (LightStone).

### Preparation of DNA from fractionated chromatin

An aliquot of each fraction corresponding to the amount of sample from 3 mg of cells was used for DNA preparation. Each aliquot was heated at 65°C overnight to reverse the crosslinking, and then successively treated with RNase A and proteinase K. Following phenol/chloroform extraction, DNA was recovered with 10 μg of glycogen by ethanol precipitation. Pellets were dissolved in 120 μl of TE [10 mM Tris (pH 7.5), 1 mM EDTA], treated with phenol/chloroform again, and then purified using a MinElute spin column (#28006, Qiagen). After elution with 30 μl of EB buffer (#19086, Qiagen), the DNA was quantified using a Quant-iT PicoGreen Kit (#P11496, Thermo). To estimate the size of the fractionated DNA, the DNA was loaded onto a 2% agarose gel and stained with SYBR Green I Nucleic Acid Stain (#50513, Lonza) and imaged using an FLA-3000G Fluorescence Imaging Analyzer (Fuji Film).

### Analyses of the proteins in fractionated chromatin

The remaining portions of the Fr-1 to Fr-3 fractions and the Fr-4 to Fr-5 fractions were 2-fold and 3-fold diluted with CDB, respectively. To recover the proteins, 100% (w/v) trichloroacetic acid (TCA) was added to the diluted fractions at a final concentration of 20%. The mixture was chilled on ice for 30 min and then centrifuged at 21 500 × g at 4°C for 20 min. After washing with ice-cold ethanol twice, the pellets were suspended in 130 μl (Fr-0), 110 μl (Fr-1), or 50 μl (Fr-2 to Fr-5) of TCA-pellet suspension buffer (TPS; 600 mM Tris (pH 8.8), 4% SDS, 8% glycerol, 0.01% bromophenol blue). To simultaneously solubilize the pellet and reverse the crosslinking, the suspension was heated at 65°C for 24 h. After centrifugation at 21 500 × g at 4°C for 10 min, the proteins were recovered in the supernatants. To observe the total protein in each fraction, the volumes of the protein preparations were adjusted with TPS among the fractions. Following treatment with 100 mM dithiothreitol (DTT) at 100°C for 5 min, the total protein was size-separated on an 8% SDS-polyacrylamide gel electrophoresis (SDS-PAGE) gel and stained with SYPRO Ruby Protein Gel Stain (#50562, Lonza). When the contents of the protein preparation were analyzed by western blotting, the preparations were adjusted among the fractions with TPS based on the amount of DNA. Following treatment with DTT, the protein preparations were loaded onto a 10% (for core histones, histone H1, GAPDH and β-actin), 8% (for HP1α, Suz12, MBD2 and MeCP2) or 6% (for BRG1) SDS-PAGE gel, subjected to electrophoresis, and then transferred to a nitrocellulose membrane (0.2 μm pore size). After blocking in 5% skim milk in Tris-buffered saline (TBS) with 0.1% Tween 20, the membranes were sequentially exposed to a primary antibody and either an alkaline phosphatase-conjugated secondary antibody or a biotinylated secondary antibody followed by streptavidin-conjugated alkaline phosphatase (#RPN1234, GE Healthcare). The membranes were developed with a BCIP-NBT Solution Kit (#03937-60, Nacalai). To prepare crosslinked proteins from the fractionated chromatin, TCA precipitants were suspended in TPS as described above, sonicated using a Sonifier liquid processor for 5 s on ice, and incubated at 4°C for at least 24 h. After centrifugation at 21 500 × g at 4°C for 10 min, the protein-containing supernatants were recovered. Following treatment with 100 mM DTT at 25°C for 30 min, the proteins were subjected to SDS-PAGE in an ice-cold electrophoresis chamber. Note that DNA is depurinated during the TCA precipitation, and subsequently digested in alkaline TPS (Supplementary Figure S5) (43). To estimate the fractional distributions of the proteins, a standard curve for quantitation was calculated from the blot signals from serially diluted samples, whose intensities were measured using ImageJ. The primary and secondary antibodies are listed in Supplementary Table S1.

### Two-dimensional (2D) electrophoresis of crosslinked proteins

Proteins were precipitated from fractionated chromatin by TCA as described above, solubilized with 20 μl of EzApply 2D Solution 2 (#AE-1435, Atto), and stored at 4°C for at least 4 days (with vigorous vortex once a day). After centrifugation at 21 500 × g at 4°C for 10 min, the proteins were recovered in the supernatant. Acetic acid-urea-tritone X-100 polyacrylamide gel electrophoresis (AUT-PAGE) was performed as described previously (44,45). Briefly, the proteins were mixed with one-tenth volume of glycerol and one-twentieth volume of 0.2% methylene blue, and loaded onto a 10% AUT-PAGE slab gel. The AUT-PAGE electrophoresis was run at a constant 1.2 W for 150 min, and the gel was sliced to obtain a gel strip in which the proteins were loaded. Gel strips were treated twice with 25 ml of SDS-exchanging buffer (62.5 mM Tris–HCl (pH 6.8), 10 mM DTT, 2.5% SDS) for 20 min, before being layered onto a 10% SDS-PAGE gel and subjected to secondary electrophoresis. As a control, recombinant histones H1.0, H3.1, H2B, and H4 (#M2501S, #M2503S, #M2505S and #M2504S, respectively, New England Biolabs) were utilized. Recombinant H1.0 and H3.1, or H2B and H4, were mixed (100 pmol each) and dispersed in 10 μl of 1% SDS in 100 mM HEPES (pH 7.6). Immediately after removal of the SDS using a HiPPR Detergent Removal Spin Column Kit (#88305, Thermo), the histones were treated with one-tenth volume of 0.5% FA at 25°C for 10 min, supplemented sequentially with one-tenth volume of 1.25 M glycine, an equal volume of EzApply 2D Solution 2, one-tenth volume of glycerol, and one-twentieth volume of 0.2% methylene blue, and subjected to 2D electrophoresis as described above. To detect histones, western blotting was performed as described in the previous section. To compare histones H1 and H3, or H4 and H2B, their signals were obtained individually, pseudo-colored red and blue, respectively, and overlaid using Photoshop (Adobe).

### Preparation and analyses of chromatin obtained from MNase-digested nuclei

HepG2 cells (50 μg wet weight) were suspended in 2 ml of Nuclei isolation buffer (15 mM Tris–HCl (pH 7.5), 60 mM KCl, 15 mM NaCl, 5 mM MgCl_2_, 0.5 mM EGTA, 300 mM sucrose, 0.5 mM 2-mercaptoethanol, 0.2% IGPAL CA-630, and a Complete Protease Inhibitor Cocktail), homogenized with 20 strokes of a Dounce tissue grinder (with tight pestle), and incubated with agitation at 4°C for 60 min. Nuclei were harvested by centrifugation at 3450 × g at 4°C for 5 min, suspended in 1 ml of MNase digestion buffer (15 mM Tris–HCl (pH 7.5), 60 mM KCl, 15 mM NaCl, 5 mM MgCl_2_, 5 mM CaCl_2_, 300 mM sucrose, 0.2% IGPAL CA-630 and a Complete Protease Inhibitor Cocktail), and homogenized with 20 additional strokes. Nuclei preparations were supplemented with 3600 gel units of MNase (#M0247S, New England Biolabs) and incubated at 37°C for 30 min, with agitation every 10 min. MNase digestion was halted by the addition of 100 μl of 100 mM EGTA. DNA was prepared from 50 μl of nuclei preparation by phenol/chloroform extraction and ethanol precipitation. The remaining nuclei were divided into two aliquots for alternative preparation methods ‘A’ and ‘B’, as summarized in Supplementary Figures S6B and C. For preparation A, nuclei were suspended in 100 μl of PBS supplemented with NaCl to a final concentration of 500 mM and agitated at room temperature for 15 min. Extracts were centrifuged at 13 800 × g at 4°C for 10 min and nucleosomes were eluted into the supernatant as reported previously (46). FA was added to the nucleosome preparation at 0.5%, and agitated at room temperature for 10 min. Crosslinking was halted by the addition of 50 μl of 1.25 M glycine. For preparation B, nuclei were suspended with 500 μl of PBS supplemented with FA at 0.5% and agitated at room temperature for 10 min. Then, 50 μl of 1.25 M glycine was added to halt the crosslinking reaction, and the nuclei were recovered by centrifugation at 3450 × g at 4°C for 5 min. To solubilize the crosslinked chromatin, nuclei were suspended in 100 μl of TSB and fragmented using a Sonifier liquid processor (at level ‘2’ for 5 s 4 times on ice). Samples were applied to a Vivaclear Mini column and the nuclear components were recovered in the flow-through. To isolate the crosslinked chromatin from preparations A and B, immunoprecipitation with an anti-H3 antibody was performed, as described above. One-thirtieth of the immunoprecipitates were subjected to western blotting following 10-fold dilution with TPS. The remaining chromatin was used for AFM observation as described above.

### Analyses of 5-methyl cytosine (5meC) in the DNA from fractionated chromatin

Two hundred ng of the DNA (adjusted to 30 μl) from each fraction was denatured by heating at 100°C for 5 min. After being immediately chilled on ice for 5 min, the DNA was spotted onto a nitrocellulose membrane (0.2 μm pore size) using a Bio-Dot Apparatus (#1706545, Bio-Rad). After the membrane was baked at 80°C for 120 min, immunoblotting with an anti-5-methyl cytosine antibody (#ab1884, Abcam) was performed as described above.

### Analyses of the DNA from fractionated chromatin

For quantitative PCR (qPCR) analyses, 500 pg (for the non-repeat sequences), 62.5 pg (for the L1 sequence), or 16.7 pg (for the *Alu* and α-satellite sequences) of the recovered DNA was used for a single reaction. To generate a standard curve, serially diluted human genomic DNA (0.76−12 500 pg; #D4642, Sigma-Aldrich) was utilized as previously described (40,47). The amount of each sequence was estimated from the respective PCR cycle threshold (Ct) value plotted on the standard curve. A 1:3 mixture of a QuantiFast SYBR Green PCR Kit (#204056, Qiagen) and a FastStart Universal SYBR Green Master Rox (#04913914001, Roche) in a 7900HT Fast Real Time PCR System (Applied Biosystems) was used for qPCR. The PCR primers are listed in Supplementary Table S2. For preparation of an NGS sequence library of the DNA from the fractionated chromatin, 28 ng of the DNA in a Crimp-cap microTUBE (#520052, Covaris) was fragmented with an LE220 Focused-ultrasonicator (Covaris). The configuration of the ultrasonication process was as follows: temperature, 7ºC; duty factor, 30%; peak incident power, 450 W; cycles per burst, 200; and time, 190 s. Following concentration via a DNA Clean & Concentrator-5 (#D4013, Zymo Research), the fragmented DNA was converted to a sequence library using a KAPA Hyper Library Preparation Kit (#KK8502, KAPA Biosystems). To analyze transcripts in the HepG2 cells, 2 μg of total RNA was converted to a sequence library using a KAPA Stranded mRNA-seq Kit (#KK8420, KAPA Biosystems). These libraries were analyzed using a HiSeq 2500 sequencer (Illumina) with the following specifications: Read1, 50 cycles.

### Bioinformatic analyses

#### Processing of RNA-sequencing reads

RNA-sequencing reads were trimmed via the fastx_trimmer function of the FASTX-toolkit (version: 0.0.14) and the last 50 bp was retained (parameter: ‘-l 50’). The transcription levels were evaluated as transcripts per kilobase million (TPM) using Sailfish (beta v0.10.0). Ensemble76 reference data with the option ‘-p 20 -l SR -r’ were employed for transcript annotation. Using this parameter, the transcription levels were categorized as follows: ‘Low’, log_10_(TPM + 1) < 0.15; ‘Mid’, 0.5 < log_10_(TPM + 1) < = 1.5; ‘High’, 2.0 < log_10_(TPM + 1).

#### Processing of DNA-sequencing reads in Fr-1 to Fr-5

The DNA sequence reads were trimmed in the same way as for RNA-seq (see *Processing of RNA-sequencing reads* paragraph). HISAT2 (version 2.0.4) was used to map the reads to the human hg38 genome using default parameters. Samtools-0.1.19 fulfilled the requirement of HISAT2. The reads employed in the analyses were qualified using Samtools (version 1.3) with ‘samtools view -q 4’. The read depth analyses were performed using ‘bam2wig.py’ in RSeQC (version 2.6.4), specifying the wigsum as 8500000000 (-t 8500000000), skipping non-unique hits reads (-u), and fixing the chromosome sizes (-s ‘hg38 chromosome size file’). To obtain the Fr-5/Fr-1 scores for the entire genome, WiggleTools (https://github.com/Ensembl/WiggleTools) was used. First, the read depth scores were scaled by the amount of recovered DNA (‘wiggletools scale’ with Supplementary Table S3), and 0.001 was added to each of the scores (‘wiggletools offset 0.001’, to avoid substitution 0 for logarithm operation (Fr-5/Fr-1)). Next, division and logarithm operations (to obtain the Fr-5/Fr-1 scores) were performed (‘wiggletools ratio’ and ‘wiggletools log 2’). A list of genes and gene predictions was downloaded from the UCSC Genome Browser (https://genome.ucsc.edu/cgi-bin/hgTables) to obtain the coordinates of the TSS regions (−250 bp to ±0 bp) for Figure 3D. Then, sequences overlapping with repetitive sequences of hg38 were eliminated using intersectBed (bedtools v2.25.0) with option -v. Repetitive sequence data were obtained from the UCSC Genome Browser (https://genome.ucsc.edu/cgi-bin/hgTables). The processed reads were employed in the following analysis except when defining Fr-5/Fr-1 scores. Python script ‘read_distrobution.py’ in RSeQC (version 2.6.4) was used to count the reads mapped to intergenic or intragenic regions, as shown in Supplementary Figures S12B and C. Hg38 genome annotation data (hg38_Gencode_V23.bed file in Sourceforge (https://sourceforge.net/p/rseqc/activity)) was used with the -r option of ‘read_distrobution.py’. Hierarchical clustering of the composition ratios of each fraction was performed using the pvclust package (https://github.com/shimo-lab/pvclust) of the R program (with the bootstrap trial time equal to 1000 (nboot = 1000)). To obtain the fractional proportions of Fr-1 to Fr-5 at the TSSs and TESs, the R script ‘ngs.plot.r’ of the ngs.plot package (https://github.com/shenlab-sinai/ngsplot) was employed. The data in Figure 5A were obtained from the results of ‘ngs.plot.r’ with the option ‘-G hg38 -R ‘genebody’’. The genes that satisfied the requirement that the ‘read count per million mapped’ values at the TSSs and TESs were larger than 0.05 (to avoid substitution 0 for logarithm operation of the Fr-5/Fr-1) were used to extract the qualified data (see Supplementary Figures S14A and B), as listed in Supplementary Table S4, and to generate the scatter plots, along with the calculated approximation lines, presented in Figure 5B.

#### Processing of MNase-seq and ChIP-Seq datasets

MNase-sequencing data were downloaded from the EMBL-EBI database (accession number: E-MTAB-1750, ERR325293). The format of the data was converted as csfastq to fastq using the Perl script ‘csfq2fq.pl’ (obtained from https://gist.github.com/pcantalupo/9c30709fe802c96ea2b3). Bowtie2 (version 2–2.3.5.1) was used to map the reads to the human hg38 genome with the default settings. A ChIP-Seq dataset (annotated to hg19) for RNAP in HepG2 cells were downloaded from the GEO database. The data were re-annotated to hg38 using the liftOver (https://genome.ucsc.edu/cgi-bin/hgLiftOver). The protocols for the analyses in Figures 5C and 6A, and Figures 5D, E and 6B were the same as those used for the analyses in Figures 5A and B, respectively.

#### Processing of Hi-C dataset

To determine the TAD coordinates of Human HepG2 cells, we employed a previous experimental dataset obtained from ENCODE (https://www.encodeproject.org/experiments/ENCSR194SRI/; library of ENCLB022KPF). Juicer algorithm (https://github.com/aidenlab/juicer/wiki) was employed to analyze interaction between genome regions and estimate TAD locations in the hg38 chromosome. HindIII was specified as the restriction endonuclease when performing juicer analysis (-y option). After performing juicer, we employed the Hi-C interaction matrix with the mapping score cut-off set to 30 and the arrowhead algorithm (https://github.com/aidenlab/juicer/wiki/Arrowhead) to estimate TAD coordinates. The following options were employed: KR norm (-k KR); sliding window size, 2000 bp (-m 2000); and ignore sparsity of the Hi-C interaction matrix (–ignore_sparsity).

Details of the procedures and commands of these bioinformatic analysis programs are provided on the public script dataset (https://bioinformatics.riken.jp/sevens-seq/Public_Script).

## RESULTS

### Chromatin was fractionated according to its local compaction state

Interactions between chromatin components, including DNA, nucleosomes, and other chromatin-related proteins, are preserved upon treatment with FA, allowing the examination of structures responsible for chromatin compaction (48). To separate compact chromatin from open chromatin, chromatin was crosslinked with FA, solubilized with SDS, fragmented by sonication, and then subjected to sedimentation velocity centrifugation on a sucrose gradient, which enables chromatin to be fractionated based on its degree of compaction (49,50) (Figure 1A). For this study, we chose to use human hepatoma HepG2 cells, for which data on epigenetic marks are available in the ENCODE database (https://www.encodeproject.org/). First, we estimated the concentration of FA needed to preserve local chromatin compaction. HepG2 cells were treated with 0, 0.25, 0.5, 0.75 or 1% FA. After centrifugation, the gradient was separated into 6 fractions (numbered ‘Fr-0’ to ‘Fr-5’ from the top to the bottom of the gradient) (Figure 1A). Following reverse crosslinking, protein and DNA components were recovered from each fraction, and analyzed by electrophoreses. Without FA treatment core histones mostly remained in Fr-0 together with other abundant proteins (‘0%’ in Supplementary Figure S1A) because SDS in the chromatin buffers would have disassembled chromatin into core histones and other proteins. With FA treatment, core histones sedimented into lower Fr-1 to Fr-5 fractions, and sedimentation velocity increased with FA concentration (arrowheads in ‘0.25%’ to ‘1%’ of Supplementary Figure S1A). This suggests that core histones in Fr-1 to Fr-5 were crosslinked with FA, and that they sedimented as large molecular weight complexes in the sucrose gradient. As shown in Supplementary Figure S1B, DNA also efficiently sedimented to lower fractions after FA treatment. When more than 0.5% of FA was used, core histones and DNA appeared in all fractions (Supplementary Figures S1A and B). When chromatin was treated with 0.5% FA, histones H3 and H1 sedimented to the fractions lower than Fr-0, while GAPDH and β-actin mostly remained in Fr-0 (Supplementary Figure S2). Thus, 0.5% FA was used for the analysis of local chromatin compaction by sedimentation velocity centrifugation.

Analysis of the fractionated DNA revealed that the average size of the DNA fragments in Fr-1 to Fr-5 was approximately 300−500 bp (Figure 1B). This suggests that chromatin in these fractions contained mainly nucleosome arrays consisting of 2–3 nucleosomes. The amount of the DNA decreased slightly in the lower fractions (Figure 1B), while histone H3 appeared to be evenly distributed across all fractions (Figure 1C). Fr-2 to Fr-5 had a similar level of H3 per unit length of DNA, indicating that a similar number of nucleosomes were present in these fractions (Figure 1D). However, H3 per unit length of DNA was lower in Fr-1 than in the other fractions, reflecting the presence of NFRs in open chromatin.

Chromatin was prepared from each fraction by immunoprecipitation with a pan anti-histone H3 antibody and then observed via HS-AFM (Figure 1E). In Fr-1, objects including histone H3 were unstructured, although some dots did appear to be gathered (arrows in ‘Fr-1’). On the other hand, bright particles were observed in Fr-2 to Fr-5. Measurement of the particle diameters revealed that the particles became larger toward Fr-5, with the median diameters ranging from 15 nm (Fr-2) to 27 nm (Fr-5) (Figure 1F). Because the length of the fractionated DNA was comparable among the fractions (Figure 1B), the larger particles likely consisted of multiple arrays of 2–3 nucleosomes and other chromatin-associated proteins. When DNA fragments in Fr-5 chromatin were treated with TdT, 3–4 DNA fragments were observed to protrude from a single particle (Supplementary Figure S3). This indicates that the particles consisted of multiple nucleosome arrays that were three-dimensionally positioned close to each other and crosslinked with FA. Considering similar levels of H3 were found in Fr-2 to Fr-5 (Figure 1D), nucleosome positioning along the DNA was unlikely to have had an effect on the size of the chromatin particles. Thus, by using our fractionation method, chromatin was successfully fractionated according to its degree of local compaction.

### Contribution of histone H1 to local chromatin compaction

**Figure 2.**
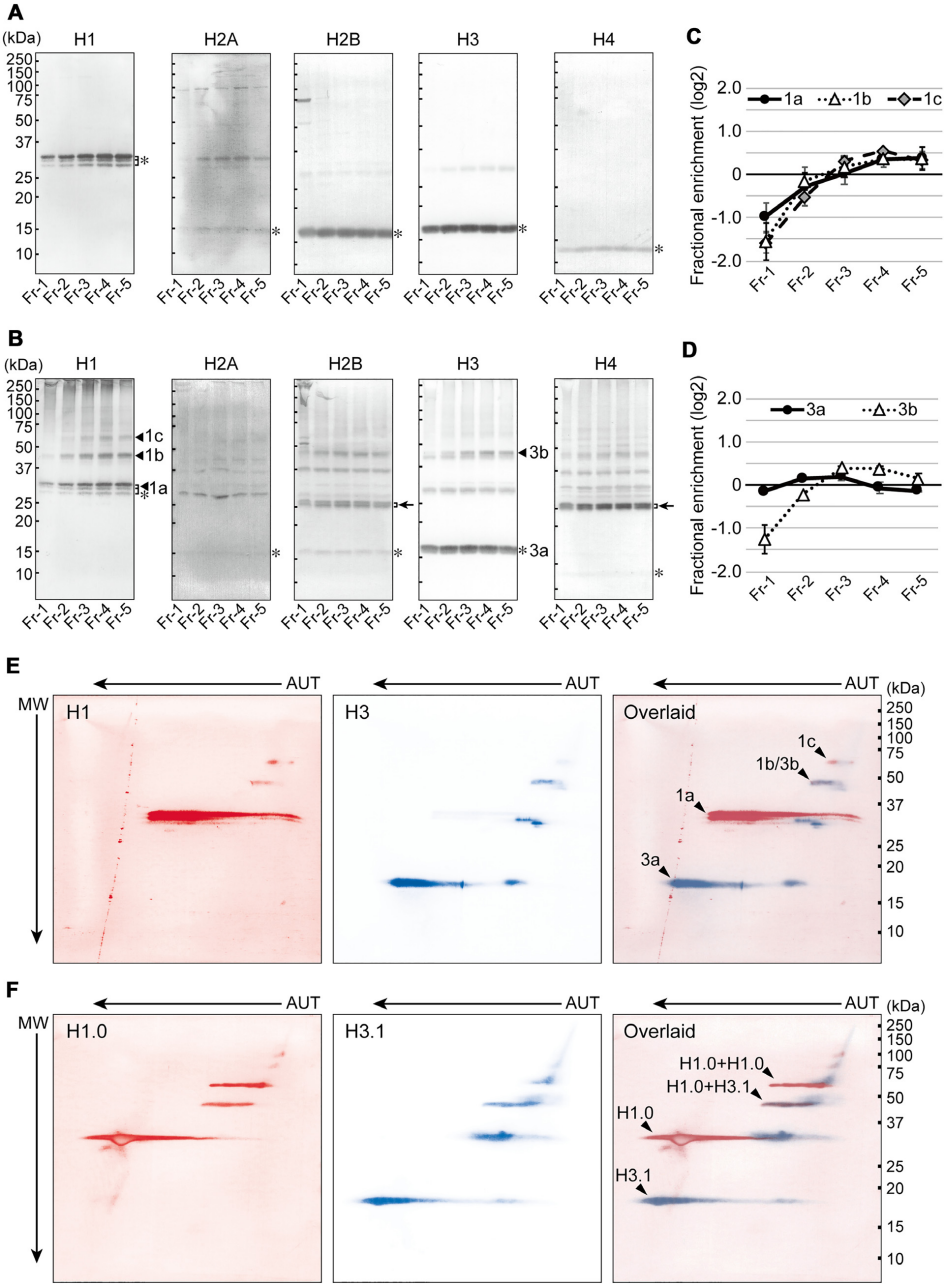
Linker and core histones in fractionated chromatin. (**A**) Following crosslink reversal, the fractionated chromatin was subjected to western blotting. Samples were normalized by DNA content prior to loading. Bands marked by asterisks are native histones. Molecular weight markers are indicated by short bars. (**B**) The fractionated chromatin without crosslink reversal was subjected to western blotting. Samples were normalized by DNA content prior to loading. Proteins that are enriched in the lower fractions are labeled with arrowheads in the ‘H1’ and ‘H3’ panels. Proteins labeled with arrows in the ‘H2B’ and ‘H4’ panels were analyzed further, as shown in Supplementary Figure S7. Bands marked by asterisks correspond to native histones. Molecular weight markers are indicated by short bars. (**C** and **D**) The distribution across the fractions of the proteins labeled with arrowheads in (B) ‘H1’ and ‘H3’, respectively. The enrichment of these proteins in each fraction is represented by the log_2_ ratio to the average. The mean and SD from at least three independent experiments are represented. (**E**) Analysis of histones H1 and H3 in crosslinked chromatin using 2D electrophoresis combining AUT-PAGE and SDS-PAGE. Pooled Fr-4 and Fr-5 was separated by 2D electrophoresis and subjected to western blotting for histones H1 and H3. Images of H1 and H3 staining were obtained individually and pseudo-colored H1 red and H3 blue (‘H1’ and ‘H3’ panels, respectively). An overlaid image of H1 and H3 is represented in the ‘Overlaid’ panel. Spots marked with arrowheads correspond to the bands labeled in (B). Molecular weight markers are indicated by short bars. (**F**) 2D electrophoresis performed as in (E) for recombinant histones H1.0 and H3.1. Arrowheads identify spots mentioned in the text. Molecular weight markers are indicated by short bars.

To clarify the molecular mechanics of chromatin compaction, protein complexes recovered from each fraction were analyzed. After precipitation with TCA and crosslink reversal, the fractionated proteins were subjected to western blotting for linker and core histones (Figure 2A). Prominent bands corresponding to native forms of each histone were observed (asterisks in Figure 2A). Importantly, linker histone H1 variants, observed as triplets, were enriched in the lower, compact chromatin fractions, while core histones appeared to be evenly distributed across all fractions (Figure 2A). The amount of H1 and H3 was calculated from their blotting intensity; the abundance of H1 was 3.2-fold higher in Fr-5 than in Fr-1, while H3 abundance in Fr-5 was l<1.5-fold the abundance in Fr-1 (Supplementary Figure S4A). When signals were normalized to the amount of H3 in each fraction, the level of H1 was 2.2-fold higher in Fr-5 than in Fr-1 (Supplementary Figure S4B).

When these experiments were performed without crosslink reversal, native histones were similarly detected across all fractions (asterisks in Figure 2B). Additional bands of less than 100 kDa, too small to be histones crosslinked to fractionated DNA (300–500 bp on average; see Figure 1B), were detected for all histones. Moreover, TCA extraction of proteins from each fraction results in the degradation of the DNA (Supplementary Figure S5; see Materials and Methods); thus these slowly migrating bands were expected to be histones crosslinked to other proteins. These bands were largely distributed evenly across all fractions, suggesting that they were not related to chromatin compaction and were crosslinked proteins within single nucleosomes, as described previously (51); however, four bands were enriched in Fr-5 (labeled 1a (as a triplet), 1b, and 1c for H1, and 3b for H3 in Figure 2B). The triplet band 1a was indistinguishable from the triplet H1 seen at approximately 30 kDa in Figure 2A, suggesting that band 1a corresponds with native histone H1. Bands 1b and 1c were estimated at 46 kDa and 60 kDa, respectively, and were enriched in the compact chromatin fractions (Figure 2C). Band 3b was similarly estimated at 46 kDa and had a similar fractionation profile as 1b (Figure 2D), raising the possibility that bands 1b and 3b may be identical. To ascertain this, 2D electrophoresis, in which AUT-PAGE and SDS-PAGE were combined, was performed on samples pooled from Fr-4 and Fr-5. Band 1b completely overlaid with band 3b (‘1b/3b’ in ‘Overlaid’ in Figure 2E), suggesting that H1 was crosslinked to H3. To investigate this hypothesis further, 2D electrophoresis was similarly performed on a mixture of recombinant histones H1.0 and H3.1 crosslinked with FA. This mixture migrated to an area close to band 1b/3b (‘H1.0+H3.1’ in ‘Overlaid’ in Figure 2F). Band 1c, above and to the right of band 1b/3b (‘1c’ in ‘Overlaid’ in Figure 2E), overlaid with recombinant H1.0 crosslinked with FA (‘H1.0+H1.0’ in ‘Overlaid’ in Figure 2F), suggesting that the 60 kDa band 1c represents the interaction between two histone H1. Note that the spots in Figure 2F tended to migrate towards the left of the blot due to random partial formylation of lysines in the unmodified, recombinant proteins. Taken together, the enrichment of bands 1a, 1b, 1c and 3b in the lower fractions suggests that compact chromatin is formed by interactions between neighboring nucleosomes via histone H1, which is anchored to histone H3 on the surface of nucleosomes.

To further examine the correlation between H1–H3 crosslinking and chromatin compaction, we next examined histone crosslinking in mono-nucleosomes, which were obtained from MNase-digested nuclei by high salt elution. In these nucleosomes, crosslinked H1 and H3 (labeled with 1b, 1c and 3b) were reduced (‘Preparation A’ in Supplementary Figure S6B) but unaffected by MNase digestion alone (‘Preparation B’ in Supplementary Figure S6B), suggesting that the H1–H3 interaction was impaired in eluted nucleosomes. Importantly, we observed dramatic differences between these preparations by HS-AFM imaging; mono-nucleosomes were identified in preparation A (Supplementary Figure S6D), while preparation B showed grape-shaped structures in which nucleosomes appeared to be gathered (Supplementary Figure S6E). Taken together, these data suggest that the interaction between H1 and H3 contributes to assembly among nucleosomes.

Previous reports have shown that the amino-terminal tail of histone H4 interacts with an acidic patch of histone H2A and H2B on an adjacent nucleosome (18,19). To evaluate the contribution of this interaction to chromatin compaction, we examined a slowly migrating doublet band that appeared to be H4 crosslinked to H2B (labeled with arrows in Figure 2B): the molecular weight of this band was ∼26 kDa, the approximate molecular wight of one H4 and one H2B molecule. The fractional distribution of this doublet was comparable in the H4 and H2B blots (Supplementary Figure S7A) and, upon 2D electrophoresis, this doublet completely overlaid (Supplementary Figure S7B), indicating direct crosslinking between these histones. In addition, the H4–H2B interaction was observed in FA-treated mono-nucleosomes (‘Preparation A’ in Supplementary Figure S6C), suggesting that the physical interaction between H4 and H2B within an individual nucleosome was preferentially observed in our method.

### The distribution of epigenetic marks and readers in fractionated chromatin

Epigenetic marks are well known to influence chromatin structure. To evaluate the contribution of the marks to the local state of chromatin compaction, we performed immunoblotting analyses for histone H3 modified at lysine residues in its amino-terminal tail. FA crosslinking was reversed and protein concentrations were normalized based on the amount of DNA prior to blotting analyses. When histone H3 lysine 9 was examined, unmodified and acetylated forms were evenly distributed throughout the fractions (‘H3K9un’ and ‘H3K9ac’, respectively, in Supplementary Figure S8A). On the other hand, tri-methylated H3 at lysine 9 was slightly enriched in Fr-2 to Fr-5 compared with its level in Fr-1 (‘H3K9me3’ in Supplementary Figure S8A and solid line in Supplementary Figure S8C). Similarly, a slight enrichment of tri-methylated H3 at lysine 27 was observed in the lower fractions (‘H3K27me3’), but the levels of the unmodified and acetylated forms did not change (‘H3K27un’ and ‘H3K27ac’, respectively, in Supplementary Figure S8A). Tri-methylated H3 at lysine 4 (H3K4me3), acetylated H4 at lysine 16 (H4K16ac), and histone H2A.Z, which are markers of open chromatin, were distributed throughout the gradient (Supplementary Figure S9). In addition, when cytosine methylation in CpG dinucleotides was assessed by dot blotting with an anti-5-methyl cytosine antibody, the signals were slightly increased toward Fr-5 (‘5meC’ in Supplementary Figure S8A and solid line in Supplementary Figure S8D). These observations suggest that H3K9me3, H3K27me3, and 5meC contribute to local chromatin compaction. To examine whether H3 is post-translationally modified in H3-H1 complexes, western blotting for H3K9me3 and H3K27me3 was performed on crosslinked chromatin. Crosslinked H3-H1 cross-reacted with both anti-H3K9me3 and anti-H3K27me3 antibodies (band 3b in Supplementary Figure S10), suggesting methylated H3 was capable of forming a complex with H1. Further analysis of this interaction showed that acetylated H3 (H3K9ac and H3K27ac) similarly crosslinked to H1 in this fraction. These observations suggest that in the lower chromatin fractions, H3 is capable of interacting with H1 regardless of its epigenetic marks.

We further investigated the fractional distribution of ‘readers’ of these epigenetic marks. The abundance of HP1α, a reader of H3K9me3, was increased toward Fr-5; however, the abundance of Suz12, a reader of H3K27me3, was decreased toward Fr-5 (‘HP1α’ and ‘Suz12’ in Supplementary Figure S8B). These observations indicate that HP1α, but not Suz12, is preferentially present in compact chromatin. When the levels of MBD2 and MeCP2, both 5meC readers, were examined, only the abundance of a small variant of MBD2, designated as MBD2b (52), was increased toward Fr-5 (‘MBD2’ and ‘MeCP2’ in Supplementary Figure S8B). When the distributions of HP1α and MBD2b were compared with those of H3K9me3 and 5meC, respectively, both readers were more highly enriched toward Fr-5 relative to the levels of the epigenetic marks (dotted vs. solid lines in Supplementary Figures S8C and D). Note that when the loaded amounts were normalized using the amount of H3, the Fr-5-biased distribution of the readers was more apparent than that of their epigenetic marks, which were evenly distributed (Supplementary Figures S8E and F). These patterns suggest that the readers are only recruited to a fraction of the available epigenetic marks before incorporation into compact chromatin.

### The distributions of active TSSs and repeat sequences in fractionated chromatin

**Figure 3.**
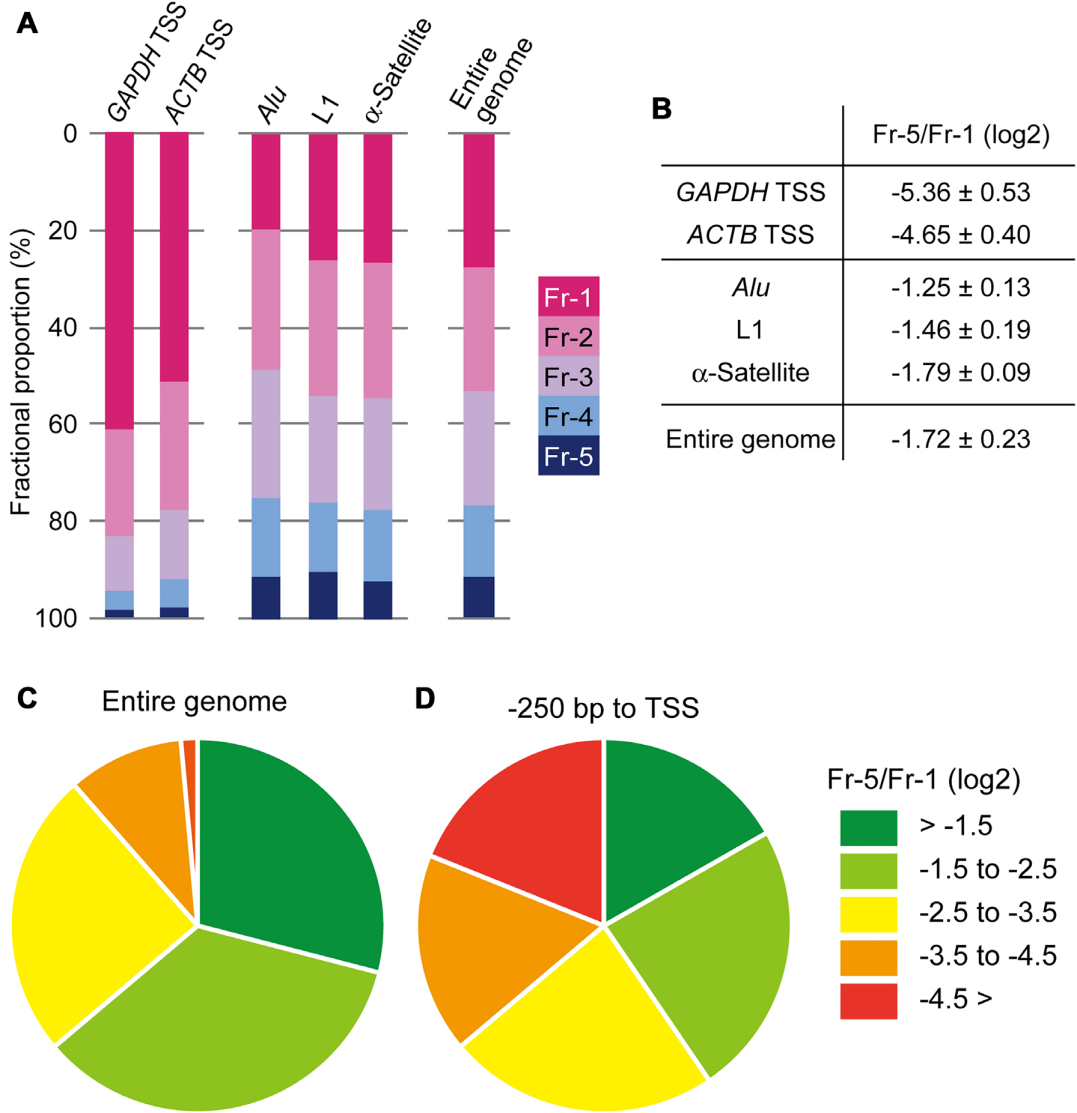
The fractional proportions and the Fr-5/Fr-1 scores of the reference regions in the genome. (**A**) The TSSs of the *GAPDH* and *ATCB* genes were analyzed as active genomic regions. *Alu*, L1, and α-Satellite repeat sequences were analyzed as repressed genomic regions. The fractional proportions of the entire genomic DNA are also shown. (**B**) The ratio of the Fr-5 proportion to the Fr-1 proportion (designated as ‘Fr-5/Fr-1’) of the genomic regions referred to in (A) was calculated and represented as a log_2_ value (the mean ± SD). Data obtained from at least three independent experiments were utilized for the calculations. (**C** and **D**) Percentages of the degrees of local chromatin compaction in the entire genome (C) and in TSS regions (–250 bp to ±0 bp) (D). The compaction degrees were classified into five groups based on the Fr-5/Fr-1 scores, as shown in the key.

Chromatin at TSSs of highly transcribed genes is opened via nucleosome eviction (11), while repeat sequences, such as transposon-derived elements, are mostly packaged into heterochromatin, which is well known to be a compact structure (53,54). To investigate how these genomic regions were fractionated by our method, their distributions were assessed using qPCR of DNA recovered from the fractions (Figure 3A). More than 50% of the TSSs for the *GAPDH* and *ACTB* genes, which are abundantly expressed in HepG2 cells, was found in Fr-1, and the proportion gradually deceased toward Fr-5, although 2% of the signal was still detected in Fr-5. As representative repeat sequences, the distributions of *Alu*, L1, and α-satellite sequences were examined by qPCR with primer pairs that annealed to conserved regions in each repeat. The proportions of these repeat sequences in Fr-1 to Fr-3 ranged from 20% to 30%, although they were lower in Fr-4 (12−15%) and Fr-5 (8−9%). These values were quite similar to the proportions of the entire genomic DNA in the fractions. This similarity is attributable to the fact that almost half of the human genome consists of such repeats. Note that when a different FA concentration was used, the fractional distributions of the specific genomic regions changed (Supplementary Figure S11A). This outcome mirrors the effect on the distributions of the entire genomic DNA (Supplementary Figure S1B). To describe simply the local state of chromatin compaction, log_2_ ratios of the proportions in Fr-5 and Fr-1 were calculated (‘Fr-5/Fr-1’ in Figure 3B). The TSSs of the *GAPDH* and *ACTB* genes showed values of –5.36 and –4.65, respectively, while the values for the repeats and entire genome ranged from –1.79 to –1.25. The apparent differences between the active and repressed reference sequences indicate that the Fr-5/Fr-1 scores are useful for representing the relative levels of the local chromatin compaction.

### Genome-wide features of local chromatin compaction

To elucidate the genome-wide features of local chromatin compaction in HepG2 cells, the DNA in each fraction was analyzed by NGS. Sequence reads were obtained as described in Materials and Methods. Approximately 90% of the reads from all of the fractions mapped to the human reference genome hg38 (Supplementary Figure S12A). All of the fractions largely consisted of intergenic and intron regions (Supplementary Figure S12B and Table S5), although the numbers of reads varied from 6.6 × 10^7^ (Fr-4) to 7.6 × 10^7^ (Fr-2) (Supplementary Figure S12C and Table S5). Hierarchical cluster analyses were performed to describe the uniqueness of Fr-1 compared with Fr-2 to Fr-5 (Supplementary Figure S12D). The Fr-5/Fr-1 scores for the entire genome were calculated from the Fr-1 and Fr-5 reads. When the chromatin compaction states were classified into five groups using the Fr-5/Fr-1 scores, approximately 90% of genomic regions were compacted at levels more than –3.5 of the Fr-5/Fr-1 scores (Figure 3C). This indicates that nearly the entire genome tends to be well compacted. However, when TSS regions were isolated, a wide variation in the Fr-5/Fr-1 scores was observed (Figure 3D). This result suggests that chromatin structure at individual TSSs fluctuate between open and compact states.

**Figure 4.**
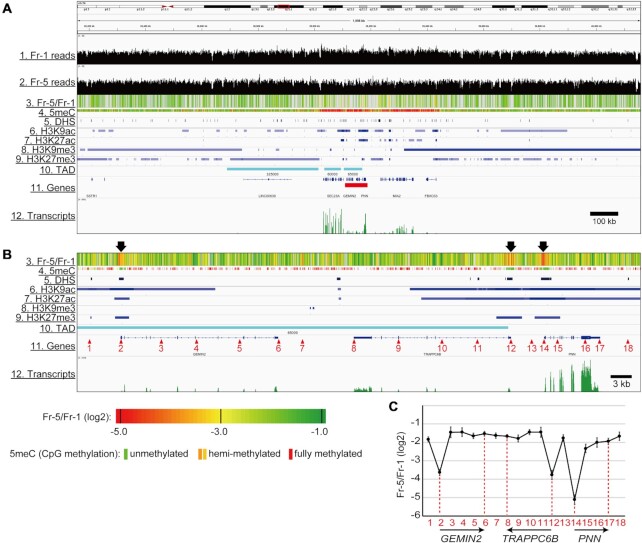
The trimmed landscape of the local chromatin compaction vs. epigenetic marks. (**A**) The Fr-5/Fr-1 magnitudes (Track 3), the epigenetic mark distributions (Tracks 4–9), TADs (Track 10) and the transcript abundance (Track 11) in a 2,000 kb region of chromosome 14 were visualized via the Integrative Genomics Viewer. (**B**) A magnified view of an 80 kb region indicated by a red bar in Track 11 of (A). The chromatin was poorly compacted in the three regions highlighted by arrows above Track 3. (**C**) The Fr-5/Fr-1 scores at the 18 positions indicated by the red numbered arrowheads in Track 11 of (B). Data obtained from at least three independent experiments are represented as the mean ± SD. The positions and orientations of the genes within the 80 kb region are shown with arrows.

Using a 2000 kb region of chromosome 14, which consists of a gene-rich region at the center and relatively long intergenic regions on both sides, the local state of chromatin compaction was compared with the levels of epigenetic marks using the Integrative Genomics Viewer (Figure 4A). The Fr-5/Fr-1 scores calculated from the Fr-1 and Fr-5 reads (Tracks 1 and 2, respectively) were represented as a heatmap (Track 3). The data for 5meC, DNase hypersensitivity (DHS), histone modifications (H3K9ac, H3K27ac, H3K9me3, and H3K27me3), and TADs in HepG2 cells were obtained from the ENCODE database (accession numbers: GSM2308630, GSM2400286, GSM733638, GSM733743, GSM1003519, GSM733754 and GSE105381, respectively) (Tracks 4–10). Transcripts from the HepG2 cells used in this study were also sequenced by NGS (Track 12). As expected from Figure 3C, there were many green stripes in Track 3 in Figure 4A, indicating that the Fr-5/Fr-1 scores at most of the positions reached nearly -1.0. This finding suggests that the chromatin across this 2000-kb region is compacted at a level similar to those of the repeat sequences, as shown in Figure 3B. While 5meC was restricted to the central gene-rich region (Track 4), H3K9me3 and H3K27me3 were mainly observed outside of the central region (Tracks 8 and 9, respectively). These distributions did not correlate with the ‘green’ regions that indicate compact chromatin (Track 3). Three TADs were observed in the 2000-kb region (Track 10), but the correlation between TADs and local chromatin compaction could not be determined because the size of these TADs (60–325 kb) was much longer than the structures represented by Fr-5/Fr-1 scores. Note that mean scores and standard deviations of Fr-5/Fr-1 within individual TADs were not remarkable compared to those of the entire genome (Supplementary Figures S13A and B), but that TAD size weakly correlated with local chromatin compaction (right panel in Supplementary Figure S13A).

Figure 4B shows a magnified view of an 80 kb region from a central part of the 2000 kb region (red bar in Track 11 of Figure 4A). Three genes, *GEMIN2*, *TRAPPC6B*, and *PNN*, appear (in this order) in the 80-kb region. The *GEMIN2* and *PNN* genes are oriented rightward, while the *TRAPPC6B* gene is oriented leftward (Track 11). As marked with arrows above Track 3 in Figure 4B, three regions with dense red stripes were found, indicating that the chromatin in these regions was poorly compacted. Intriguingly, these regions were located over the TSSs of the three genes (Tracks 3 versus 11) and corresponded to stretches without 5meC (Tracks 3 versus 4). These observations suggest that the absence of 5meC might be required for the local openness of chromatin at TSSs. The three regions also corresponded to stretches with DHS (Tracks 3 versus 5), indicating that the open chromatin can be well digested by DNase I. H3K9ac, H3K27ac and H3K27me3 were recruited over the TSSs but spread more widely compared with their distributions in the ‘red’ regions (Tracks 3 versus 6, 7 and 9, respectively). Again, the local chromatin compaction appeared to be distinct from any structures defined by these histone modifications. *TRAPPC6B* TSS with red stripes overlapped with the right boundary of the TAD (Tracks 3 versus 10). In the entire genome, a quarter of TAD boundaries formed in relatively open chromatin showing Fr-5/Fr-1 scores less than –2.5 (inner pie chart in Supplementary Figure S13C).

The fractional distributions at 18 points (marked with red numbered arrowheads in Track 11 of Figure 4B) were evaluated by qPCR (Supplementary Figure S11B). More than 40% of the TSSs of the three genes was found in Fr-1 (Points 2, 12 and 14), while the proportions of the other points in Fr-1 were <30%. The Fr-5/Fr-1 score of each point was calculated (Figure 4C). The score at the *PNN* TSS was –5.12 (Point 14), which was lower than those of the *GEMIN2* TSS (–3.63) and the *TRAPPC6B* TSS (-3.77) (Points 2 and 12, respectively). These scores were inversely correlated to the transcript abundance, as shown in Track 12 in Figure 4B. The regions outside the TSSs showed much higher Fr-5/Fr-1 scores (–2.33 to –1.44) (Figure 4C), which were comparable to the scores of the repeat sequences (Figure 3B). These results suggest that chromatin is *per se* compacted without making a distinction between repeat and non-repeat sequences; however, the compaction is locally attenuated at the TSSs of active genes.

**Figure 5.**
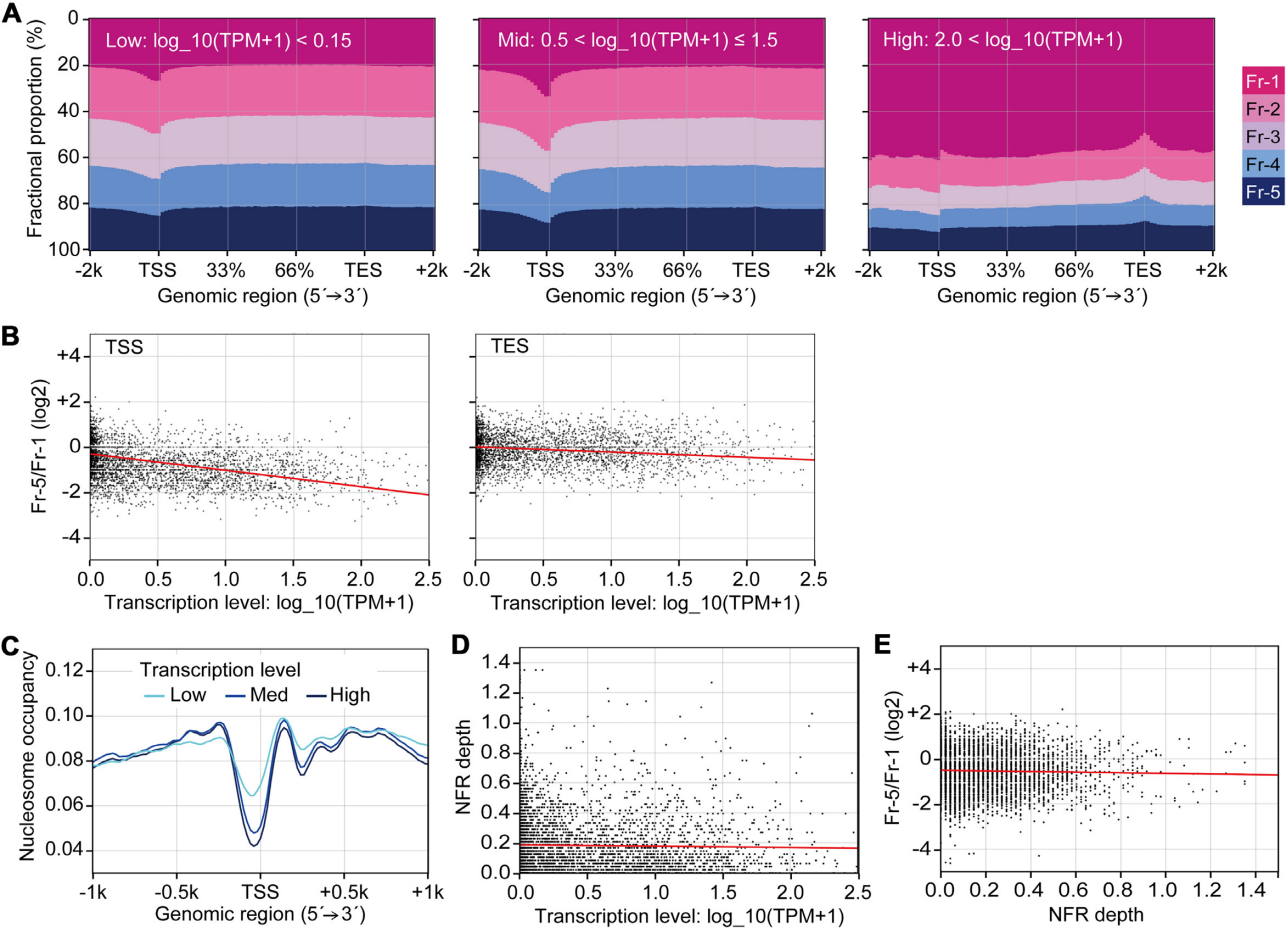
Genome-wide comparison between local chromatin compaction and transcription levels. (**A**) The fractional proportions from 2 kb upstream of the TSSs to 2 kb downstream of the TESs of active genes. The genes were divided into three groups based on their transcription levels (values of log_10_(TPM + 1)). (**B**) Scatter diagrams comparing the transcription levels and the Fr-5/Fr-1 scores at the TSSs or TESs of active genes. The red line in each panel represents an approximation line of the scatter points. The correlation coefficients (*r*) for TSS and TES are –0.4329 and –0.1703, respectively. Data for each gene are listed in Supplementary Table S4. (**C**) Nucleosome occupancy in the TSS regions (–1 kb to +1 kb) of active genes. The genes were divided to three groups as described in (A). (**D**) A scatter diagram comparing the transcription levels and NFR depth (the distance between the lowest and highest nucleosome levels within the TSS region (–250 bp to ±0 bp)). The red line represents the line of best fit for the scatter points. The correlation coefficient (*r*) is –0.0243. (**E**) A scatter diagram comparing NFR depth and Fr-5/Fr-1 scores. The red line represents the line of best fit for the scatter points. The correlation coefficient (*r*) is –0.0294.

We next focused on the bodies of active genes. To clarify the relationship between transcription level and local chromatin compaction, active genes were categorized into three groups based on their transcription levels (‘Low’, ‘Mid’ and ‘High’ in Figure 5A). Nearly the entire gene bodies of the ‘Low’ genes were evenly detected in all of the fractions, while only the TSSs were more abundant in Fr-1 and less abundant in Fr-5. This pattern was also observed for the ‘Mid’ genes. Importantly, the Fr-1-biased distribution of the ‘Mid’ TSSs was more apparent than that of the ‘Low’ TSSs, suggesting an inverse correlation between the compaction at the TSS and the transcription level. On the other hand, for ‘High’ genes, their entire bodies were largely detected in Fr-1, perhaps because the multiple RNAPs required to transcribe simultaneously the same gene during highly active transcription lead to nucleosome eviction (55). Intriguingly, the transcription end sites (TESs) of the ‘High’ genes were slightly less abundant in Fr-1 compared with the levels of the other parts of the gene bodies. Thus, moderate compaction of the TESs may be part of the transcription termination mechanism for the ‘High’ genes as described previously (56,57). Next, the Fr-5/Fr-1 scores of the TSS and TES of each gene were calculated and plotted on scatter diagram against the transcription level. The data used for the calculations were restricted as described in Materials and Methods and in Supplementary Figures S14A and B. The downward slopes of the approximation lines for both sites indicated an inverse correlation between the Fr-5/Fr-1 scores and the transcription levels (Figure 5B). The slope of the TSS line was steeper than that of the TES line (see correlation coefficients in the figure legend), indicating that the correlation of the TSSs was stronger than that of the TESs. We also analyzed nucleosome positioning in HepG2 cells from MNase-sequencing data in the EMBL-EBI database (accession number: E-MTAB-1750). The patterns of nucleosome occupancy around the TSS showed an NFR corresponding to a ‘nucleosome valley’ (Figure 5C). Consistent with previous reports (12–15), a correlation between the depth of the NFR valley and the transcription level was clear when the average positioning was calculated from multiple TSSs in each group classified according to their transcription level (Figure 5C). However, for individual genes, the depth of the valley, which was calculated as the distance between the lowest and highest nucleosome occupancy within the TSS region (–250 bp to ±0 bp), was not correlated with the transcription level (Figure 5D), indicating that transcription from TSSs with shallow NFR valley depths (<0.2) was relatively high. This suggests that the absence of nucleosomes on TSSs is not necessarily a determinant for enhanced transcription, although there is a possibility that non-histone proteins occupy such TSSs (58). The NFR depth was also not correlated with the Fr-5/Fr-1 scores (Figure 5E), suggesting that nucleosome eviction does not robustly influence the local chromatin compaction state.

**Figure 6.**
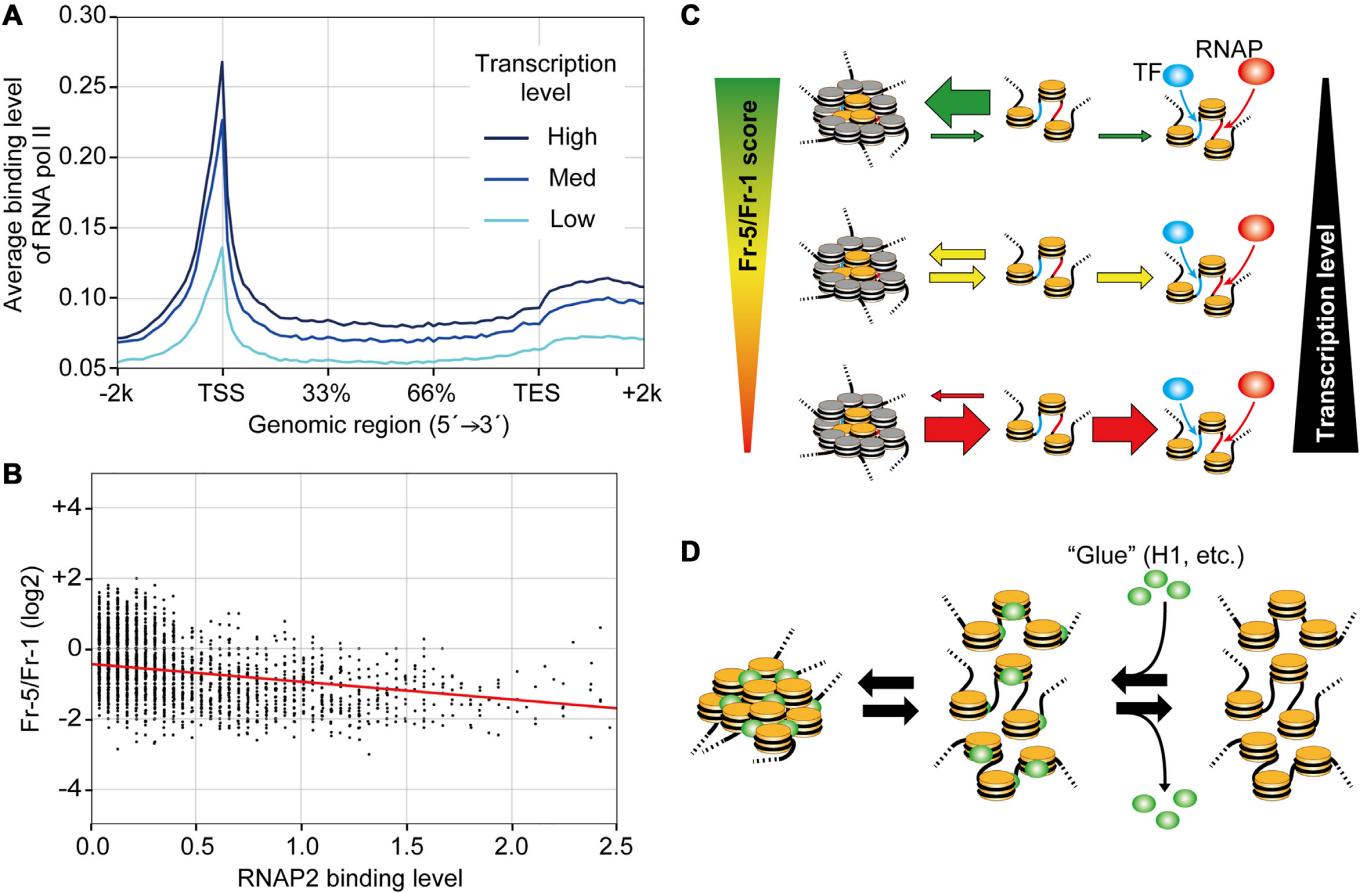
A model of the quantitative regulation of transcription by the local chromatin compaction state via the binding frequency of RNAP and TFs. (**A**) The binding levels of RNAP from 2 kb upstream of the TSSs to 2 kb downstream of the TESs of active genes. The genes were divided into three groups as described in Figure 5A. (**B**) A scatter diagram comparing the RNAP binding levels and the Fr-5/Fr-1 scores at the TSSs. The red line represents an approximation line of the scatter points. The correlation coefficient (*r*) is –0.2109. (**C**) The transcription level is regulated by the equilibrium between local chromatin compaction and openness, which affects the binding frequency of RNAP (red ellipse) and TF (blue ellipse) to TSS (red line) and enhancer (blue line), respectively. When the equilibrium is biased toward the compact state with a high Fr-5/Fr-1 score, transcription is attenuated because of the shortened window of time allowed for RNAP and TF bindings. Conversely, a bias toward the open state with a low Fr-5/Fr-1 score widens this window and increases the level of transcription. Nucleosomes along RNAP and TF binding sites are colored yellow, while other nucleosomes are colored gray. This model is shown as animations in Supplementary Figure S15. (**D**) Chromatin tends to become compact upon loading of ‘glue’ proteins, such as histone H1 (green ellipses), as these proteins physically link adjacent nucleosomes.

To search for a correlation between the compaction at the TSS and RNAP binding, data from a chromatin immunoprecipitation-sequencing (ChIP-Seq) experiment for RNA polymerase II (RNAP2) in HepG2 cells were obtained from the Gene Expression Omnibus (GEO) database (accession number: GSM2864932). Expectedly, the binding level of RNAP2 peaked at the TSSs, and the peak heights were reflected in the transcription levels (Figure 6A). In a scatter diagram of the RNAP2 binding levels against the Fr-5/Fr-1 scores at the TSSs, an inverse correlation was observed (Figure 6B), indicating that RNAP2 binds less frequently to TSSs in chromatin with higher Fr-5/Fr-1 scores. Taken together, these results suggest that local chromatin compaction, particularly at TSSs, can attenuate transcription possibly by reducing the binding frequency of RNAP.

## DISCUSSION

In the field of chromatin biology, the positioning of nucleosomes, which occupy approximately 147 bp of DNA, has been well characterized by NGS combined with MNase treatment (59,60). On the other hand, TADs consisting of megabase-scale DNA loops have been identified by the Hi-C technique (32–35). In this study, sedimentation velocity centrifugation in combination with NGS revealed chromatin compaction of array(s) consisting of 2–3 nucleosomes that could represent another hierarchical level of chromatin organization.

DNA-processing enzymes, for example, endonuclease or transposase, have been historically used to analyze chromatin structure (61). This type of analysis is based on whether the enzyme can access and then digest (or recombine with a probe sequence) the linker DNA between nucleosomes. Thus, these approaches are based on the accessibility rather than the inaccessibility of chromatin. However, in chromatin compacted within the narrow space of a few nucleosomes, the linker DNA may not be hidden among the nucleosomes, and it may be accessible to enzymes that are smaller than the RNAP complex (62). It has been reported that endonucleases such as MNase and *Alu*I restriction enzyme can equally digest genomic DNA in open and compact chromatin, although low concentrations of such enzymes are useful for identifying hyper-accessible regions (63,64). In this study, to simultaneously evaluate the accessibility and inaccessibility of chromatin, a strategy was designed to biochemically separate open and compact chromatin via fractionation by ultracentrifugation (Figure 1A). As confirmed by the HS-AFM experiments (Figure 1E and F), chromatin was successfully fractionated according to the magnitude of its local compaction. Because this compaction is achieved based on inter-nucleosomal interactions, the absence or presence of NFRs must directly influence the formation of compact chromatin. Nevertheless, nucleosomes were evenly distributed per unit length of DNA across the fractions (Figure 1D). Figure 5E also shows that there was no correlation between Fr-5/Fr-1 scores and NFRs. Thus, local chromatin compaction appears to occur independently of the nucleosome level.

Considering the fluctuation in the movement of nucleosomes (29,37,38), chromatin must exist as a dynamic equilibrium between self-associated and dissociated nucleosome states. In this study, chromatin would have contained a mixture of variously compacted structures representing the different chromatin equilibriums in the cell mixture at the time of cell harvesting. This could be the reason why highly active TSSs and repeat sequences did not sediment to any one particular fraction, but were distributed throughout the fractions (Figure 3A). The proportion of compact vs. open chromatin in a mixed cell population is reflected in the Fr-5/Fr-1 score. Interestingly, the observation of high Fr-5/Fr-1 scores throughout nearly the entire genome (Figure 3C) leads us to imagine that chromatin throughout the nucleus is locally compacted. In addition, fluctuation in the movement of nucleosomes (29,37,38) suggests that this compaction accompanies dynamic ‘breathing’. Local assembly of nucleosomes during the breathing may contribute to the packing of nucleosomes within irregular aggregates (29–31) and to sub-loops of TADs (65). The degree of compaction in TADs is consistent throughout the genome (Supplementary Figures S13A and B), suggesting that compacted structures can be formed both inside and outside TADs (Supplementary Figure S13D). The extent of inter-nucleosomal interactions may determine the formation of TADs together with their boundary components. The equilibrium in chromatin compaction restricts the time window during which RNAP can access TSSs, suggesting that the length of this window could determine RNAP binding frequency (Figure 6C). The length of this window also affects the binding frequency of transcription factors (TFs) (Figure 6C). The inverse correlation between transcription levels and Fr-5/Fr-1 scores at TSSs (‘TSS’ in Figure 5B) could be interpreted as follows: When the equilibrium is shifted toward compact chromatin with a high Fr-5/Fr-1, transcription would be attenuated because of less RNAP binding; when the equilibrium is shifted toward open chromatin with a low Fr-5/Fr-1 score, transcription would be elevated because of more RNAP binding (Figure 6C) (see animations in Supplementary Figure S15).

Histone H1 is enriched, per unit amount of histone H3, in the lowest fractions following sedimentation velocity centrifugation (Supplementary Figure S4B), suggesting that the recruitment of histone H1 is limited to a subset of nucleosomes, which compose compact chromatin. Importantly, in the compact chromatin, H1 is positioned proximal to another H1 since H1 molecules appeared to crosslink to each other following treatment with FA (Figure 2E). A similar crosslinking of H1 has been reported previously (66,67). Using cryo-electron microscopy experiments, a similar compaction of *in vitro*-reconstituted chromatin by recombinant H1 was observed (68). Moreover, a single molecule of H1 has been shown to promote the condensation of a tetranucleosome array (69). It is possible that inter-nucleosomal interactions through H1 could drive this chromatin compaction. H1 repeatedly associates with and dissociates from chromatin every few minutes (70,71). This dynamic association of H1 could keep chromatin compaction in equilibrium. All of the epigenetic marks examined in this study, i.e., H3K9ac, H3K27ac, H3K9me3, H3K27me3 and 5meC, were detected throughout all of the fractions, although their specific fractional distributions varied slightly (Supplementary Figure S8A). These observations indicate that none of these marks are a direct determinant of whether or not chromatin is compacted. However, considering that HP1α and MBD2b were more abundant toward Fr-5 than were H3K9me3 and 5meC (Supplementary Figures S8C–F), some H3K9me3 and 5meC marks might have served as platforms for HP1α and MBD2b, respectively. The level of HP1α and MBD2b peaked in Fr-5, while H1 began to plateau at Fr-3 (Supplementary Figures S8C–F vs. Supplementary Figures S4A and B), suggesting that HP1α and MBD2b may be incorporated to chromatin already bound by H1, allowing HP1α to bridge two adjacent nucleosomes (72). Although the structural relationship between MBD2b and nucleosomes remains unclear because MBD2 family proteins bind directly to DNA, it is clear that histone H1 and HP1α operate on a local structure that comprises a few nucleosomes of chromatin. Together, histone H1, HP1α, and MBD2b could function as ‘glue’ between the adjacent nucleosomes in compact chromatin (Figure 6D). On the other hand, chromatin remodeling factors (CRFs) may play a role in the opening of local chromatin, because the amount of BRG1 increased toward Fr-1 (Supplementary Figure S9).

Heterochromatin and euchromatin, which were originally defined via cytological observations, have been proposed as intranuclear structures that act as a transcriptional switch (73). Furthermore, epigenetic marks have been widely recognized to influence the formation of heterochromatin and euchromatin (74). Our study provides evidence for the chromatin structure that is determined by its degree of local compaction. Because a portion of the H3K9me3 and 5meC marks, which are considered heterochromatin marks, were involved in local chromatin compaction via recruitment of HP1α and MBD2b, respectively, the compact chromatin may be an intermediate structure in the process leading to the formation of typical heterochromatin. Fine-tuning of transcription would be achieved at such a flexible level in the structural hierarchy of chromatin.

## DATA AVAILABILITY

NGS sequence library datasets and Fr-5/Fr-1 scores in HepG2 cells have been submitted to the NCBI Gene Expression Omnibus (GEO) (http://www.ncbi.nlm.nih.gov/geo/) under accession number GSE146632. A link to the Big Bed file is available on the UCSC genome browser on our website (https://bioinformatics.riken.jp/sevens-seq/UCSC/public.bb). In-house scripts can similarly be accessed on our website (https://bioinformatics.riken.jp/sevens-seq/Public_Script/).

## Supplementary Material

gkab587_Supplemental_Files
