# Supplementary material for: Local states of chromatin compaction at transcription start sites control transcription levels
Source: Nucleic Acids Res. 2021 Jul 7;49(14):8007–23. doi: 10.1093/nar/gkab587 (PMC8373074; doi:10.1093/nar/gkab587)
Supplement: gkab587_Supplemental_Files [file gkab587_supplemental_files.zip › FigS15.pptx]

## Slide 1
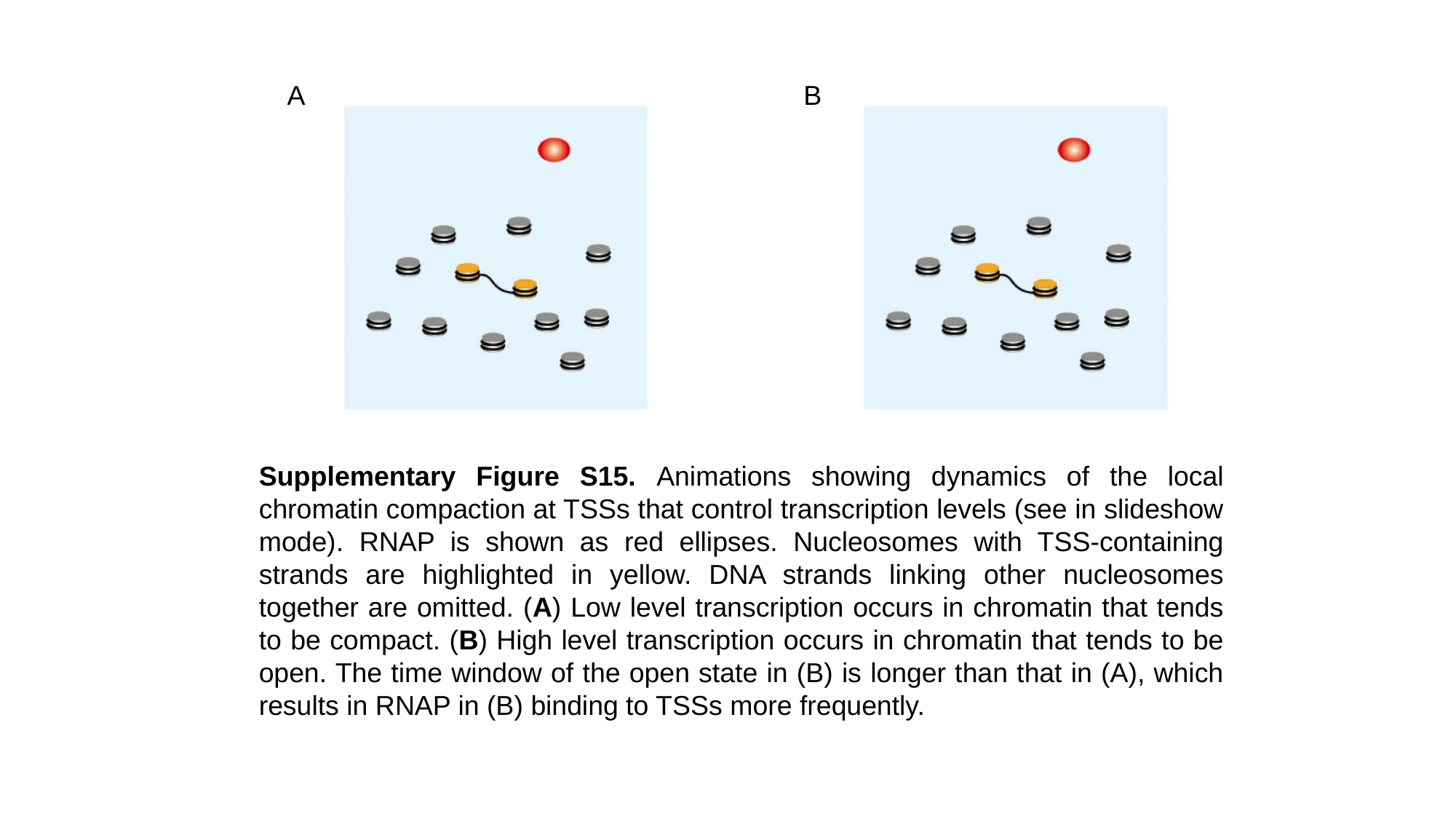

A
B
Supplementary Figure S15. Animations showing dynamics of the local chromatin compaction at TSSs that control transcription levels (see in slideshow mode). RNAP is shown as red ellipses. Nucleosomes with TSS-containing strands are highlighted in yellow. DNA strands linking other nucleosomes together are omitted. (A) Low level transcription occurs in chromatin that tends to be compact. (B) High level transcription occurs in chromatin that tends to be open. The time window of the open state in (B) is longer than that in (A), which results in RNAP in (B) binding to TSSs more frequently.
