# Supplementary material for: Local states of chromatin compaction at transcription start sites control transcription levels
Source: Nucleic Acids Res. 2021 Jul 7;49(14):8007–23. doi: 10.1093/nar/gkab587 (PMC8373074; doi:10.1093/nar/gkab587)
Supplement: gkab587_Supplemental_Files [file gkab587_supplemental_files.zip › FigS1–14.pdf]

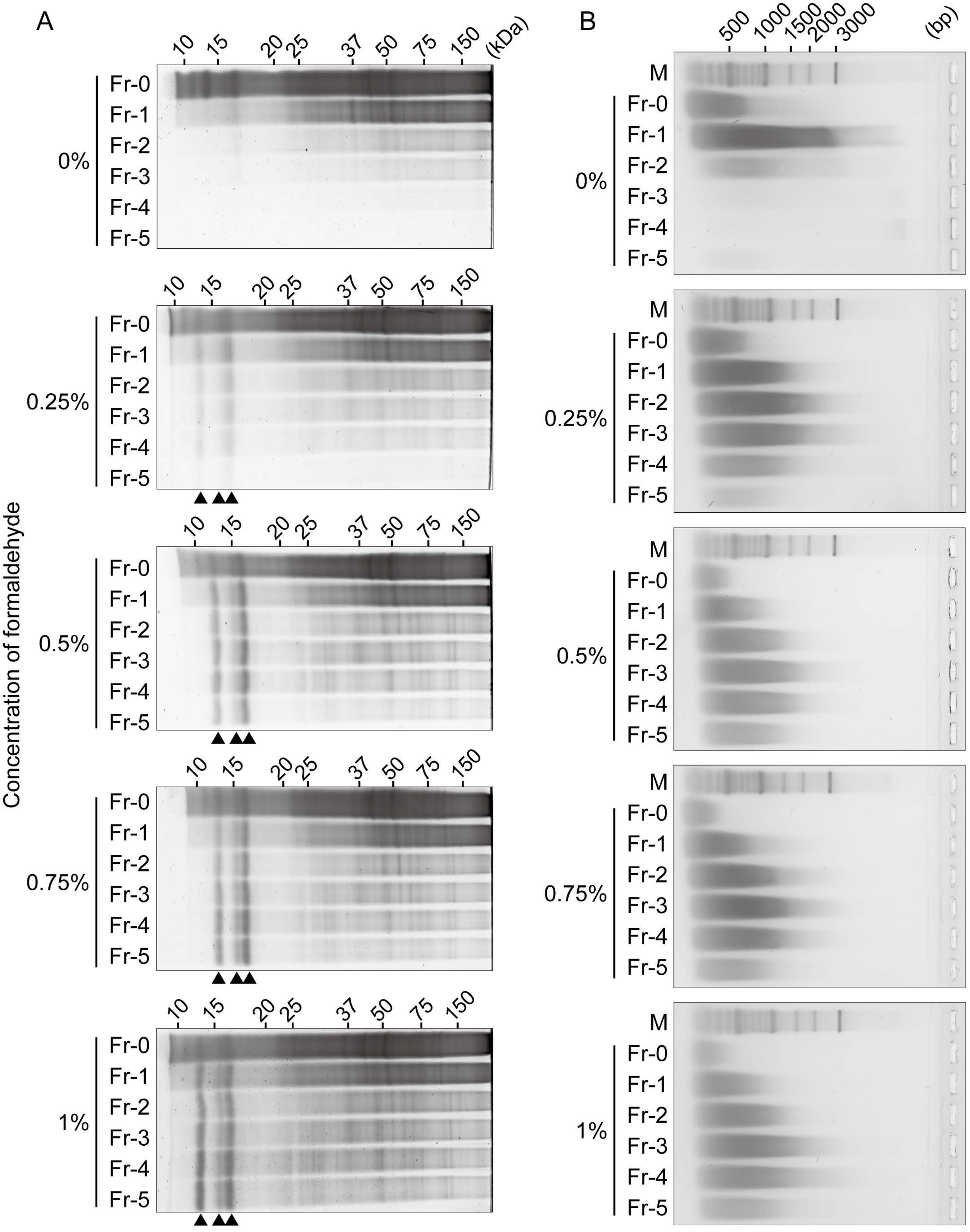

Supplementary Figure S1. The size-distribution of protein and DNA components prepared from chromatin that had been fractionated by sedimentation velocity centrifugation after crosslinking using various FA concentrations. (**A**) The proteins were size-separated on a 10% SDS-PAGE gel and stained by SYPRO Ruby. Molecular weight markers are indicated by short bars. Arrowheads represent core histones. (**B**) The DNA was size-separated on a 2% agarose gel and stained by SYBR Green I. In all panels, the same size-markers (indicated with “M) were used.

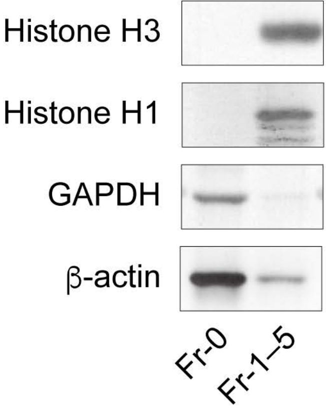

Supplementary Figure S2. Comparison between the protein components in the fraction that remained in the uppermost layer (Fr-0) and those in the fractions that sedimented into the sucrose gradient (Fr-1–5). Fr-1–5 were collected individually, and merged into a single tube. After the volumes of the uppermost layer Fr-0 and the merged fractions Fr-1–5 were adjusted to obtain equal volumes, proteins in the same volume of sample were separated by SDS-PAGE and detected by western blotting.

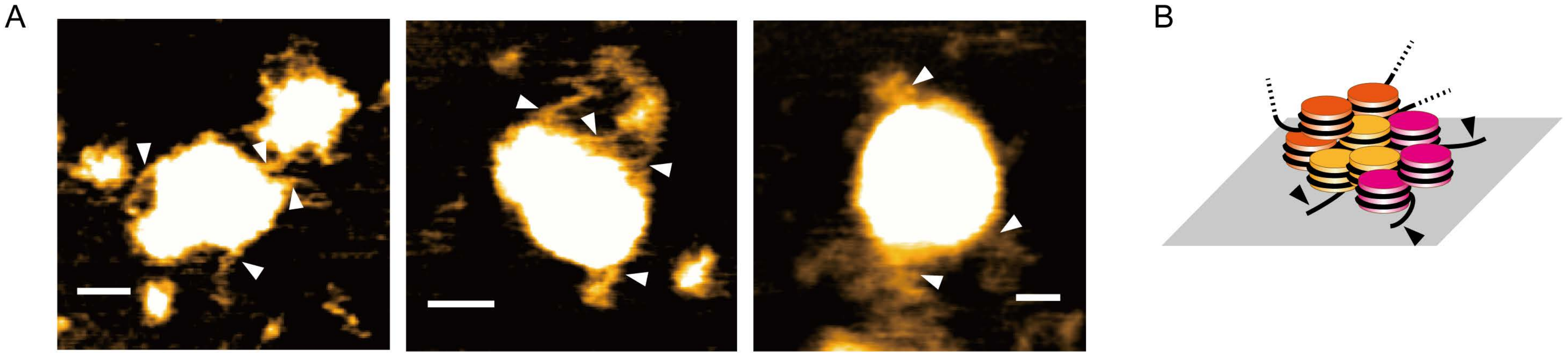

Supplementary Figure S3. Fr-5 chromatin particles consist of multiple nucleosome arrays. (**A**) After treatment of Fr-5 chromatin with TdT, extended DNA strands were observed using HS-AFM, which revealed that more than three DNA fragments protruded from single particles (arrowheads). This indicated that at least two nucleosome arrays were present in Fr-5 chromatin. Scale bars are 20 nm. Hydrophobic aggregation was avoided by observation under 1% SDS following elution of chromatin from the immunoprecipitates (see MATERIALS AND METHODS). (**B**) Schema of the chromatin particles in (A). When three arrays of three nucleosomes (colored in orange, yellow, and pink in each array) compose a single chromatin particle, the arrays will have six termini. In AFM analyses, some of DNA fragments extending from the termini were attached to the mica surface (gray sheet), and observed as protruded structures (arrowheads).

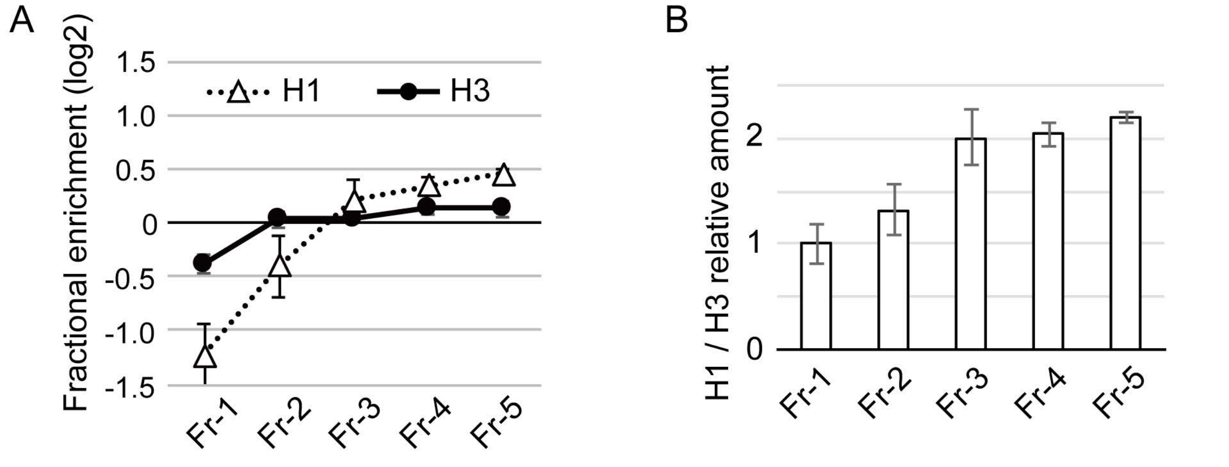

Supplementary Figure S4. Enrichment of histone H1 in compact chromatin. (**A**) The fractional distribution of histones H1 and H3 was evaluated from western blot band intensities and is represented as the log2 ratio to the average. For H1, the upper band in the triplet was measured. Data obtained from at least three independent experiments are represented as the mean ± SD. (**B**) After normalizing the loaded samples among the fractions based on the amount of histone H3, the amount of histone H1 in each fraction was represented as its amount relative to that in Fr-1 (=1). Data were obtained from at least three independent experiments, and represented as the mean ± SD.

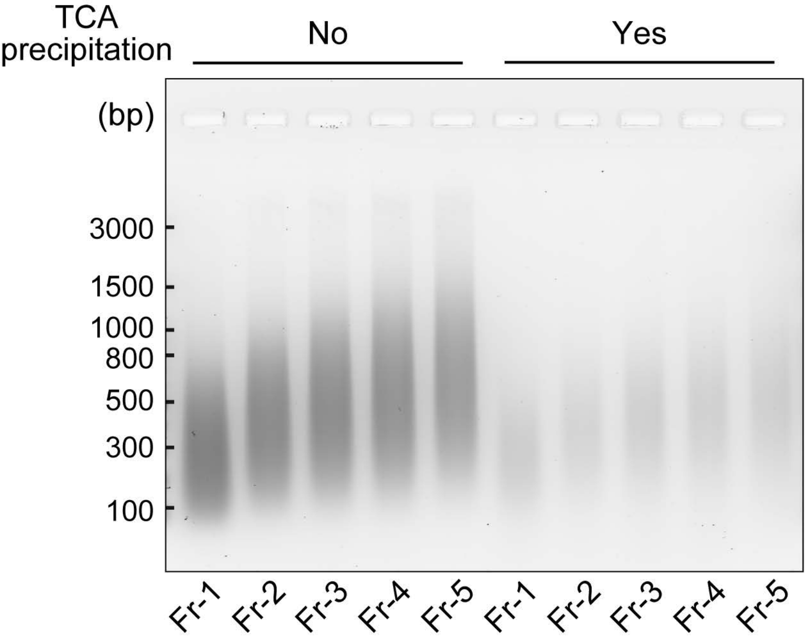

Supplementary Figure S5. Degradation of DNA in fractionated chromatin during TCA precipitation. DNA samples prepared with or without TCA extraction from equal volumes of fractionated chromatin (labeled “Yes” or “No” , respectively) were applied to a 2% agarose gel and stained with SYBR Green I.

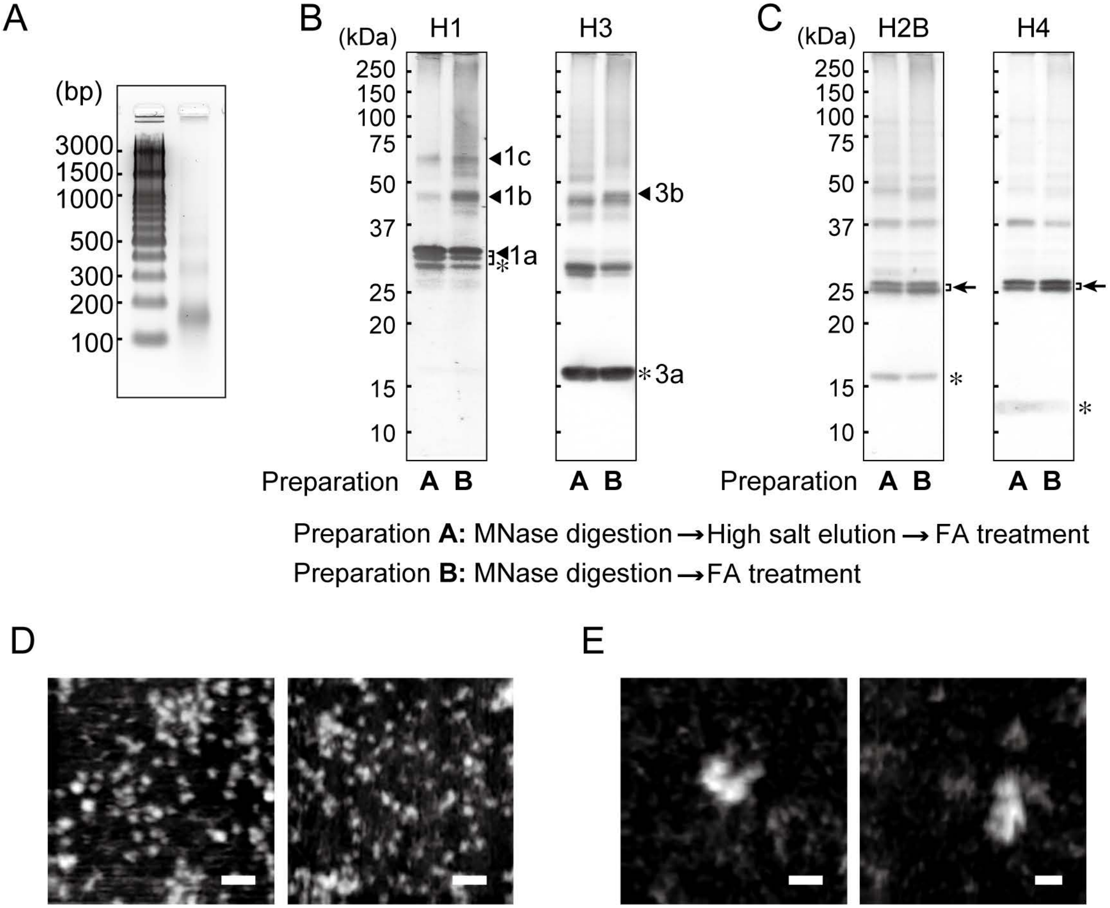

Supplementary Figure S6. Analysis of chromatin prepared from MNase-digested nuclei. (**A**) DNA treated with MNase was separated by size on a 3% agarose gel. A major band around 150 bp indicates that chromatin was most likely dispersed as mono-nucleosomes. (**B** and **C**) The FA-crosslinking pattern of H1 and H3 (B), and H2B and H4 (C), was analyzed by western blotting. Samples were prepared using the methods described below the images: for preparation A, chromatin was eluted from MNase-digested nuclei as mono-nucleosomes with high salt PBS, and treated with FA; for preparation B, MNase-digested nuclei were directly treated with FA, before chromatin was prepared. (**D** and **E**) HS-AFM images of chromatin in preparation A (D) and B (E). Scale bars represent 30 nm.

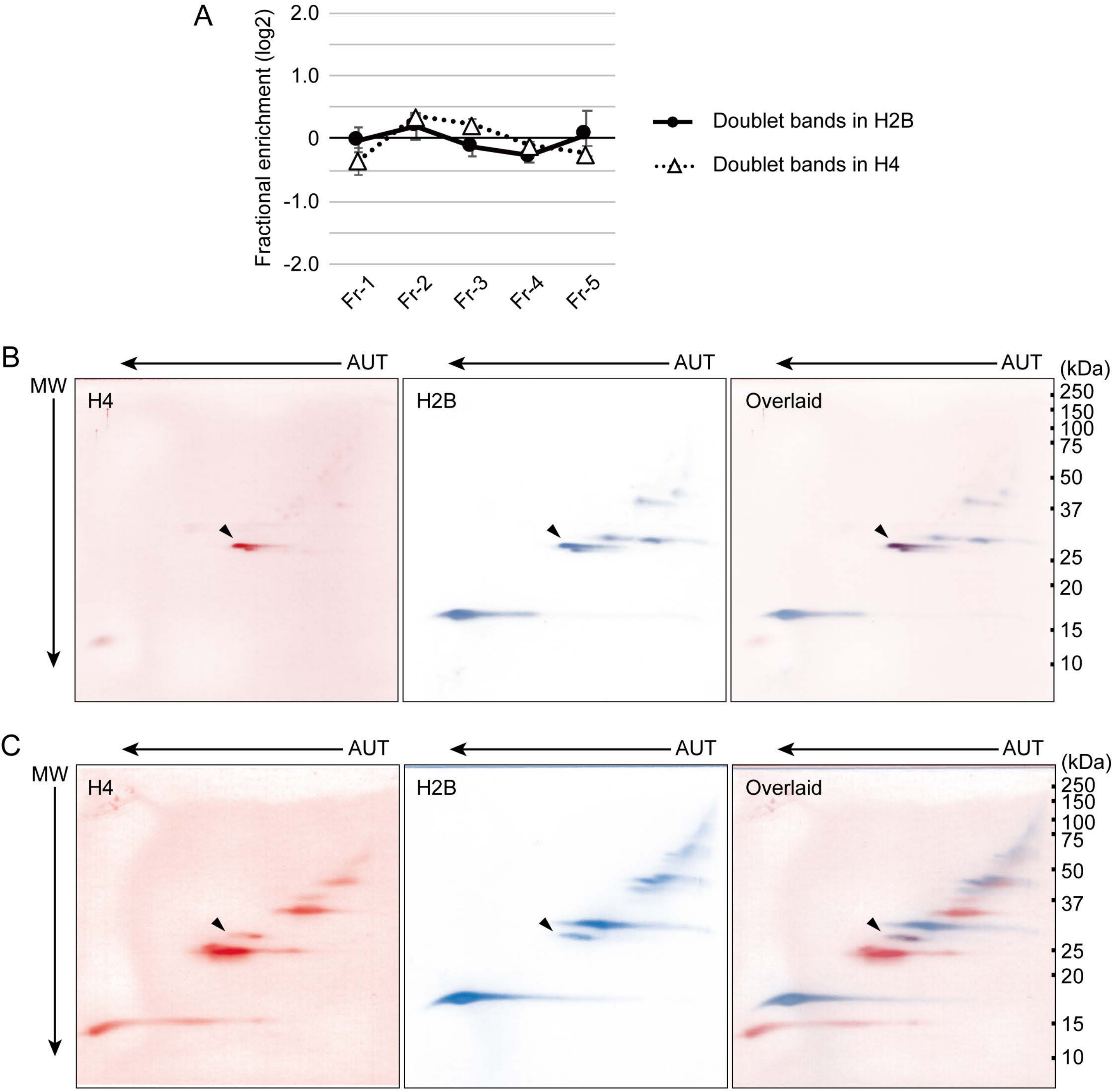

Supplementary Figure S7. Analysis of H2B and H4 crosslinks labeled with arrows in Figure 2B. (**A**) The fractional distribution of crosslinked H2B and H4 was calculated from the intensity of the doublet bands indicated with arrows in Figure 2B, and protein enrichment is represented by the log2 ratio to the mean. The mean and SD from at least three independent experiments are represented. (**B**) Analysis of crosslinked histones using 2D electrophoresis combining AUT-PAGE and SDS-PAGE. Samples pooled from all fractions were separated by 2D electrophoresis and subjected to western blotting for H4 and H2B. Blotting signals for H4 and H2B were obtained individually and pseudo-colored red and blue, respectively. An overlaid image is represented in the "Overlaid" panel. Spots marked with arrowheads correspond to crosslinked H4-H2B. Molecular weight markers are indicated by short bars. (**C**) 2D electrophoresis performed as in (B) for recombinant histones H4 and H2B. Arrowheads represent spots corresponding to crosslinked H4-H2B. Molecular weight markers are indicated by short bars.

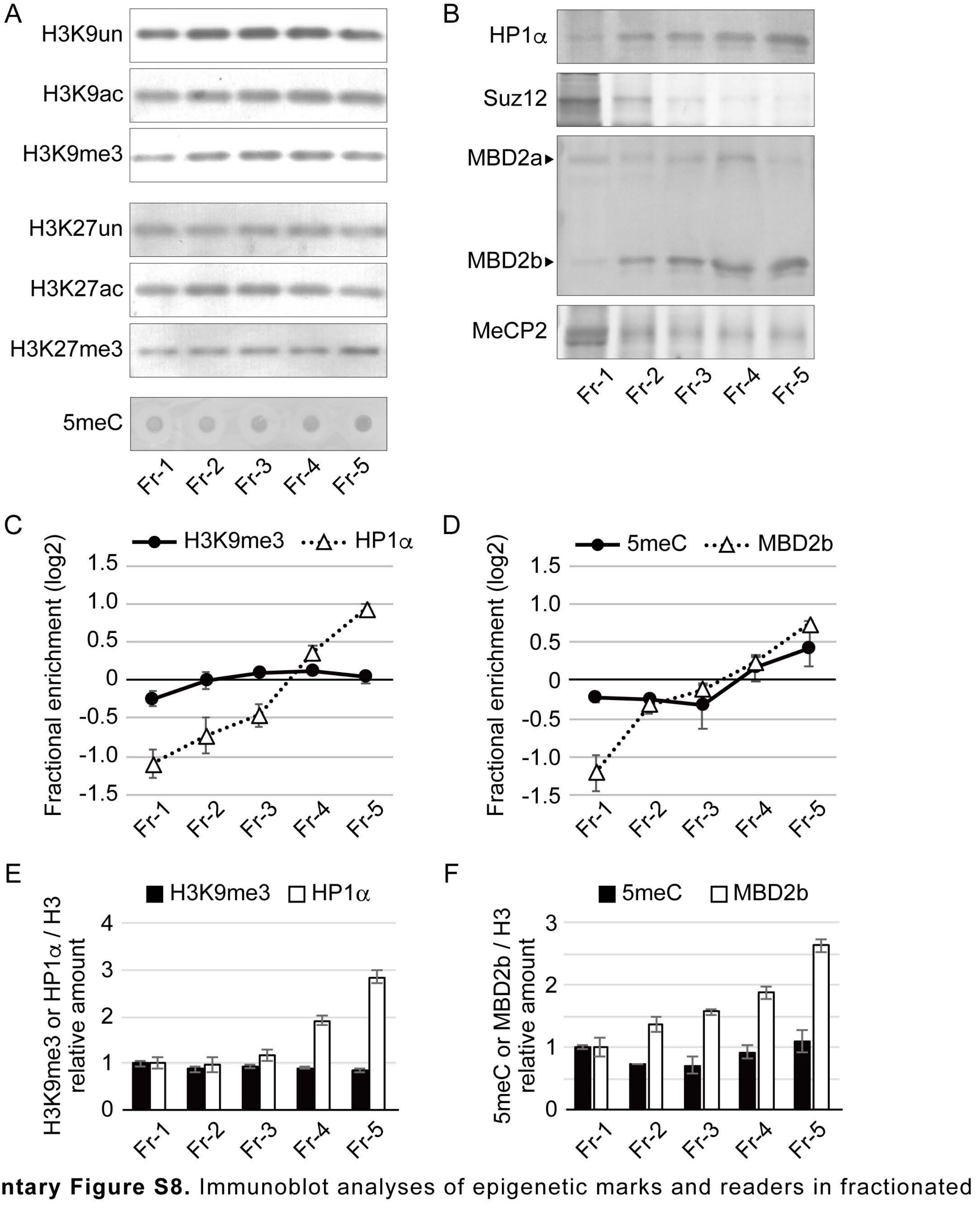

Supplementary Figure S8. Immunoblot analyses of epigenetic marks and readers in fractionated chromatin. (**A**) The fractional distribution of the epigenetic marks was analyzed by western blotting. For 5meC, DNA from the fractionated chromatin was spotted onto a membrane and analyzed by dot blotting. (**B**) The fractional distribution of the epigenetic readers was analyzed by western blotting. The large and small variants of MBD2, designated MBD2a and MBD2b, respectively, are marked by arrowheads. (**C** and **D**) The fractional distribution of epigenetic marks and the proteins that bind them. The relative enrichment of H3K9me3 and HP1α (C), and 5meC and MBD2b (D) was calculated from the intensity of the blot signals and is represented by the log2 ratio to the average. Data obtained from at least three independent experiments are represented as the mean ± SD. (**E** and **F**) After normalizing the loaded samples among the fractions based on the amount of histone H3, the amounts of chromatin components mentioned in (C) and (D), respectively, in each fraction were represented as their amounts relative to those in Fr-1 (=1). Data was obtained from at least three independent experiments, and represented as the mean ± SD.

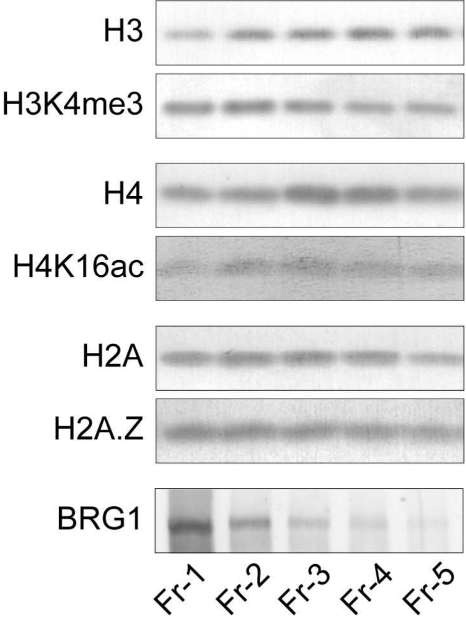

Supplementary Figure S9. Fractional distribution of chromatin components related to chromatin openness. Using western blotting, the distribution of H3K4me3, H4K16ac, H2A.Z, and BRG1 was analyzed. The distribution of H3, H4, and H2A are included as controls.

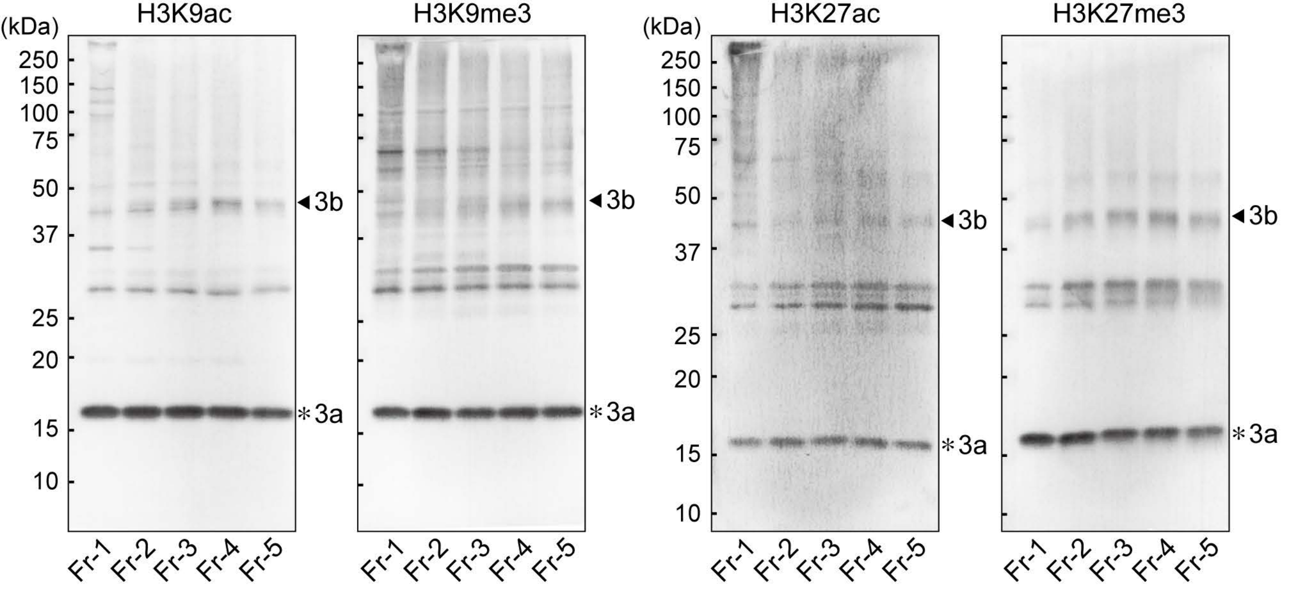

Supplementary Figure S10. Crosslinked proteins in fractionated chromatin were subjected to western blotting with antibodies against post-translationally modified H3. Bands corresponding to native and H1-crosslinked H3 were labeled with 3a and 3b, respectively, as in Figure 2B. Molecular weight markers are indicated by short bars.

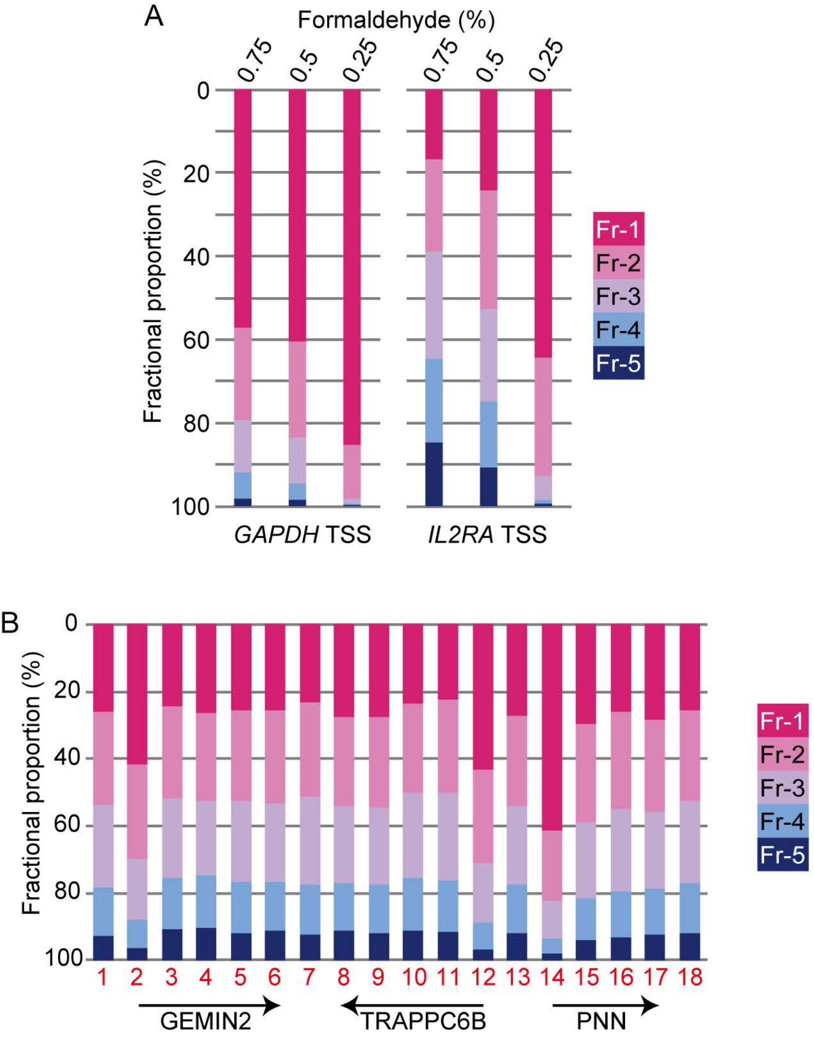

Supplementary Figure S11. qPCR analyses of DNA in the fractionated chromatin. (**A**) The fractional proportions of the TSSs of GAPDH (active) and IL2RA (repressed) genes in the fractionated chromatin following treatment with different FA concentrations. (**B**) The fractional proportions of the 18 positions marked by red numbered arrowheads in Figure 4B. The positions and orientations of the three genes within the 80 kb region are shown as arrows.

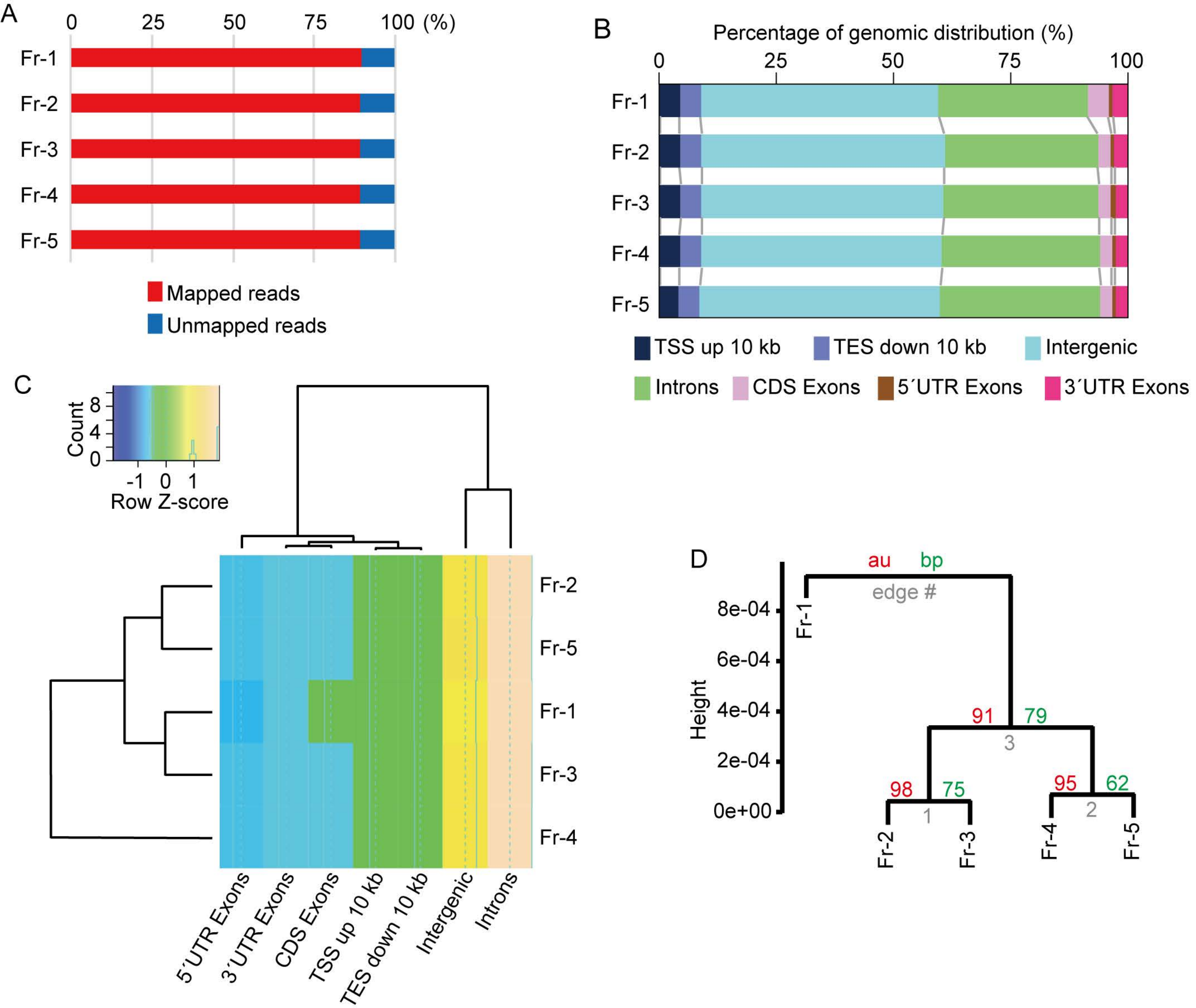

Supplementary Figure S12. Annotation analyses of the NGS reads. (**A**) The percentages of the mapped vs. unmapped reads in each fraction. Mapping of the NGS reads to the human hg38 genome was performed as described in MATERIALS AND METHODS. (**B**) The reads were annotated as being within 10 kb upstream of the TSS (TSS up 10 kb), within 10 kb downstream of the TES (TES down 10 kb), intergenic regions, introns, cds exons, 5´ UTR exons, or 3´ UTR exons in the human hg38 genome. (**C**) Classification analyses of the number of the NGS reads (in rows) vs. the genomic annotations, as shown in (B) (in columns). (**D**) Hierarchical cluster analyses of the composition ratio of each fraction. The composition according to genomic annotations is shown in (B). The red “au” and green “bp” values are p-values of approximately unbiased and bootstrap probability, respectively.

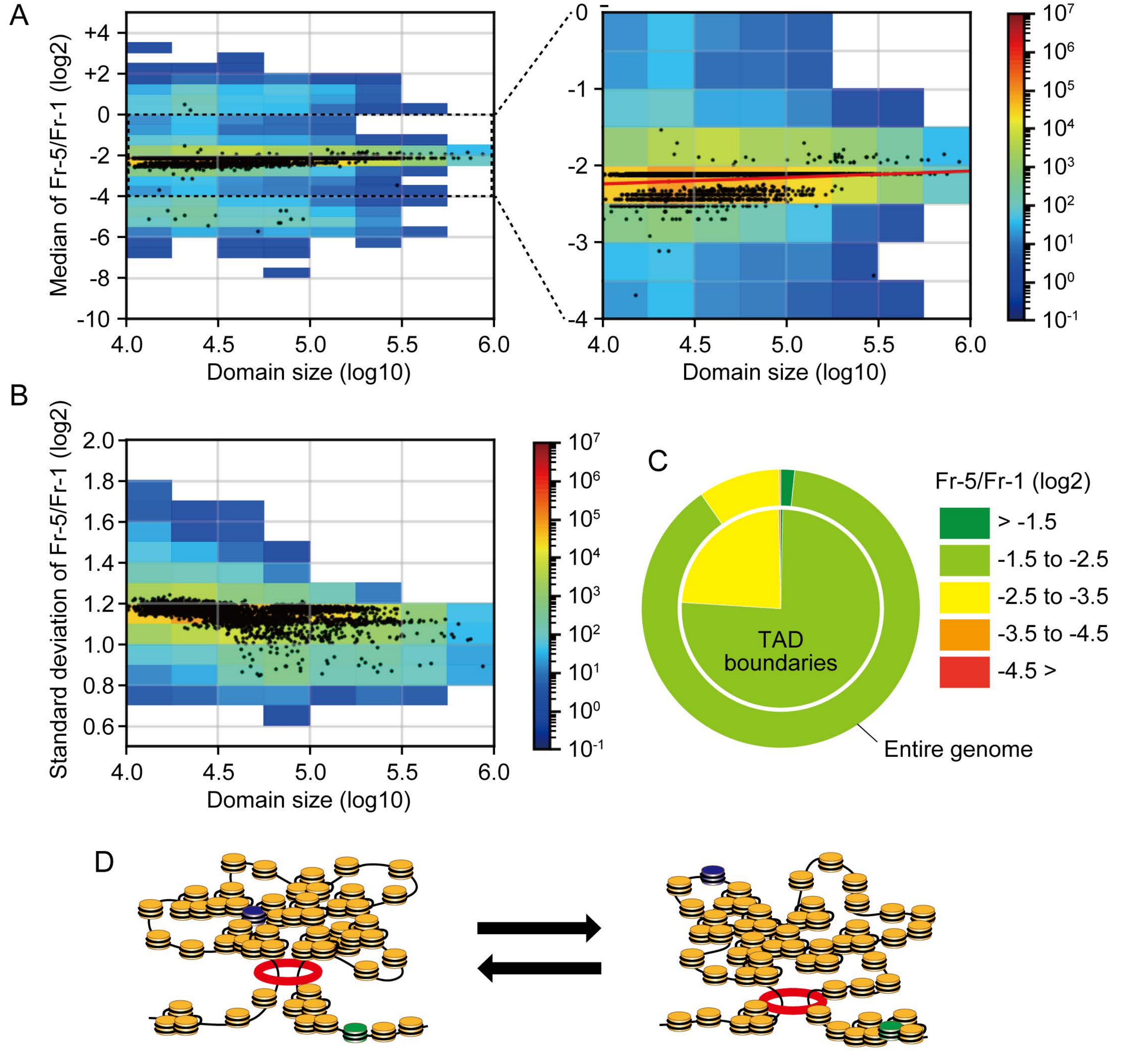

Supplementary Figure S13. Local chromatin compaction in TADs. (**A**) Median values of Fr-5/Fr-1 scores (log2) within individual TADs are plotted as black dots on a colored background to show the distribution of Fr-5/Fr-1 scores over the entire genome. Because TADs varied in length, the number of Fr-5/Fr-1 scores within a single TAD was used as the domain size for the X-axis of the graphs. The background Fr-5/Fr-1 scores were calculated as median values of Fr-5/Fr-1 picked up from the entire genome randomly. Their distribution frequency is expressed as jet-colored gradation. The area marked by the dotted square in the left panel is magnified in the right panel. The red line in the right panel is the line of best fit for the medians of TADs. The correlation coefficient (r) is 0.2748. (**B**) Standard variation values of Fr-5/Fr-1 scores (log2) within individual TADs are plotted as black dots on a colored background representing the entire genome as described above. (**C**) Median values of Fr-5/Fr-1 scores (log2) around boundaries of TADs (inner pie chart). Medians of Fr-5/Fr-1 scores within 1 kb-size regions centered around TAD boundaries were used as representative values of the boundaries, and classified into five groups as shown in the key. For comparison with the entire genome, 1 kb-size regions were picked up from the entire genome randomly, and their medians were calculated (outer doughnut chart). (**D**) The local chromatin compaction occurs regardless of whether TADs are formed or not. In other words, a nucleosome both inside a TAD (blue-colored) and outside a TAD (green-colored) would switch between compaction and openness repeatedly from moment to moment. A TAD boundary is represented by a red ring.

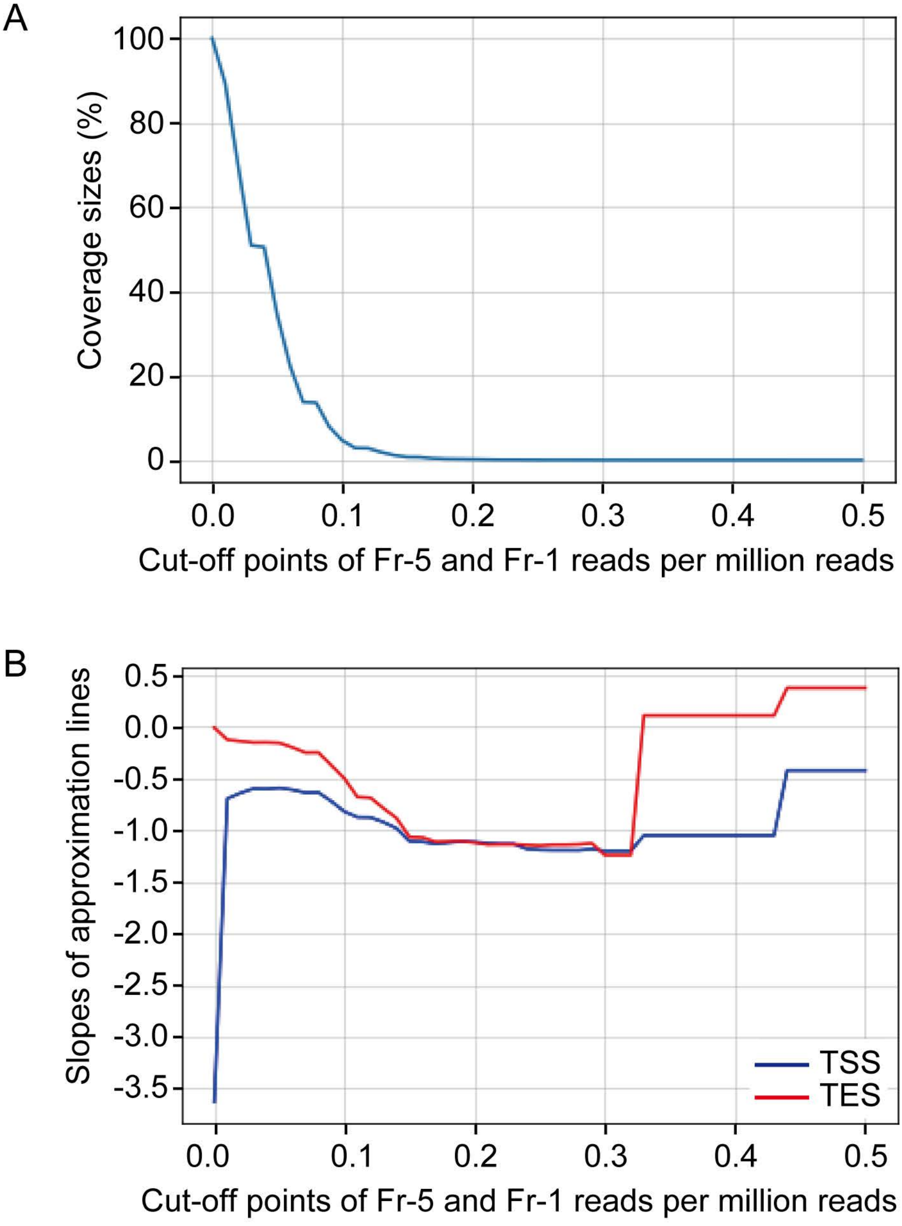

Supplementary Figure S14. Establishing a cut-off point for the Fr-5 and Fr-1 reads (per million mapped reads) for the scatter diagrams in Figure 5. 0.05 was chosen as the cut-off point to avoid substitution 0 in the logarithm operation for the Fr-5/Fr-1 scores. (**A**) The relationship between the cut-off points and coverage sizes of the sequenced reads. When a cut-off of 0.05 was used, approximately 40% of the reads were still covered. (**B**) The relationship between the cut-off points and the slopes of the approximation lines in scatter diagrams showing the transcription levels vs. the Fr-5/Fr-1 scores at TSSs and TESs (Figure 5B with a cut-off point of 0.05).
